# Exploring the Gas‐Phase Formation and Chemical Reactivity of Highly Reduced M_8_L_6_ Coordination Cages

**DOI:** 10.1002/anie.202212710

**Published:** 2022-10-07

**Authors:** Michael C. Pfrunder, David L. Marshall, Berwyck L. J. Poad, Ethan G. Stovell, Benjamin I. Loomans, James P. Blinco, Stephen J. Blanksby, John C. McMurtrie, Kathleen M. Mullen

**Affiliations:** ^1^ Centre for Materials Science (CFMS) Queensland University of Technology (QUT) 2 George Street Brisbane Queensland 4000 Australia; ^2^ School of Chemistry and Physics Queensland University of Technology 2 George Street Brisbane Queensland 4000 Australia; ^3^ Central Analytical Research Facility (CARF) Queensland University of Technology 2 George Street Brisbane Queensland 4000 Australia

**Keywords:** Coordination Cage, Electron Transfer, Low Oxidation State Transition Metals, Mass Spectrometry, Supramolecular

## Abstract

Coordination cages with well‐defined cavities show great promise in the field of catalysis on account of their unique combination of molecular confinement effects and transition‐metal redox chemistry. Here, three coordination cages are reduced from their native 16^+^ oxidation state to the 2^+^ state in the gas phase without observable structural degradation. Using this method, the reaction rate constants for each reduction step were determined, with no noticeable differences arising following either the incorporation of a C_60_‐fullerene guest or alteration of the cage chemical structure. The reactivity of highly reduced cage species toward molecular oxygen is “switched‐on” after a threshold number of reduction steps, which is influenced by guest molecules and the structure of cage components. These new experimental approaches provide a unique window to explore the chemistry of highly‐reduced cage species that can be modulated by altering their structures and encapsulated guest species.

## Graphical abstract

Coordination cages containing porphyrin subunits are reduced up to fourteen times in the gas phase. While the presence or absence of Nickel(II) coordinated in the porphyrin binding site does not affect the rate of reduction, the reactivity of the reduced species toward molecular O_2_ is significantly enhanced in the former case. Additionally, the presence of a C_60_ fullerene guest within the nickelated cage reduces its reactivity.

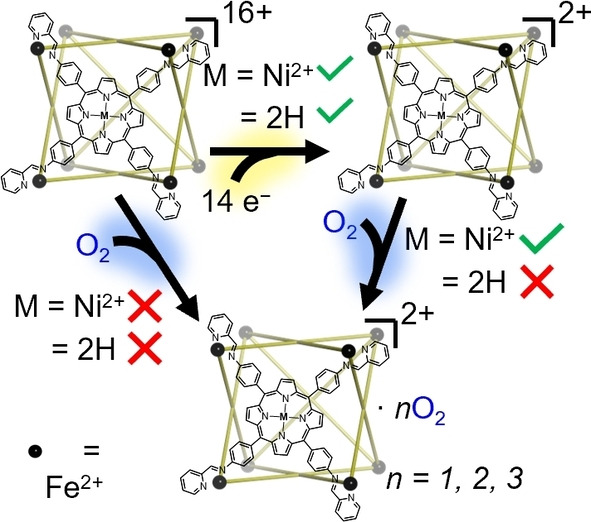

## Introduction

Transition‐metal complexes with accessible low oxidation states are fundamental to modern catalysis.^[1]^ Their incorporation into porous supramolecular architectures such as cages, macrocycles and metal‐organic frameworks is a proven strategy for producing “tailor‐made” catalysts for certain target reaction products owing to associated confinement and cooperative effects,^[2]^ and in some cases switchable catalytic behavior,^[3]^ and remains an area of significant scientific opportunity.^[4]^ Metalloporphyrins are well known for their rich redox chemistry^[5]^ which can manifest coordinatively unsaturated, soft transition‐metal sites capable of performing catalysis^[6]^ and as a result have been widely used in this approach.^[7]^ Confinement effects arising through their incorporation into cages has enabled the selective functionalization of fullerenes,^[8]^ a process with significant implications in the field of perovskite solar cells.^[9]^

This area of research continues to receive considerable attention and while significant advancements have been made, the procurement and analysis of highly reduced supramolecular species in liquid solution remains a challenge. This is due to the difficulty in obtaining pure samples of supramolecular species which instead often exist in equilibrium within a dynamic library of reaction products, as well as the occurrence of irreversible structure degradation during electrochemical manipulations.

An alternate method of investigating the reduction of ionic supramolecular species without complication is to carry out the required electrochemical manipulation in the gas phase using a mass spectrometer. This approach is advantageous over solution‐based methods due to its ability to yield, analyze and probe the properties of multiply reduced forms of compounds in the absence of competing reagents or solvents that would otherwise hinder the study of the intrinsic properties of the substrate.^[10]^ Whilst these experiments are confined to the gas phase, the findings may inform on the properties of highly reduced compounds in condensed phases, such as components in metal‐organic frameworks, where such highly reduced species may be stabilized.^[11]^ Initially developed for sequencing peptides and proteins,^[12]^ this technique involves a chemically induced single electron transfer (ET) to a multiply‐charged cation, such as a coordination cage, thus forming a charge‐reduced positive ion. In the context of protein sequence analysis, intact charge‐reduced cations are undesirable, however there is increasing interest in harnessing these electron transfer reactions to generate and characterize cationic metal complexes in uncommon low oxidation states. Charge‐reduced helices and metallosquares with up to 4 metal centres (e.g., Cu, Ag, Fe, Pt) have been investigated.^[13]^ The tandem mass spectrometry (MS^n^) capabilities of ion trap mass analyzers enable re‐isolation and further interrogation of the properties of each intact charge‐reduced product ion, rather than analyzing an averaged ensemble of all species present.^[14]^ For example, Munshi and co‐workers have studied the gas‐phase vibrational spectra of mono‐nuclear nickel, copper, and ruthenium complexes in the +1 oxidation state after reduction from the initial +2 state generated by electrospray ionization.^[15]^ Parker and Gronert compared the gas‐phase reactivity of [M(phen)_2_]^2+^ and reduced [M(phen)_2_]^+^ complexes (M=Fe, Co, Ni, Cu, Zn, phen=1,10‐phenanthroline) toward carbon‐halide bond activation.^[16]^

**Figure 1:**
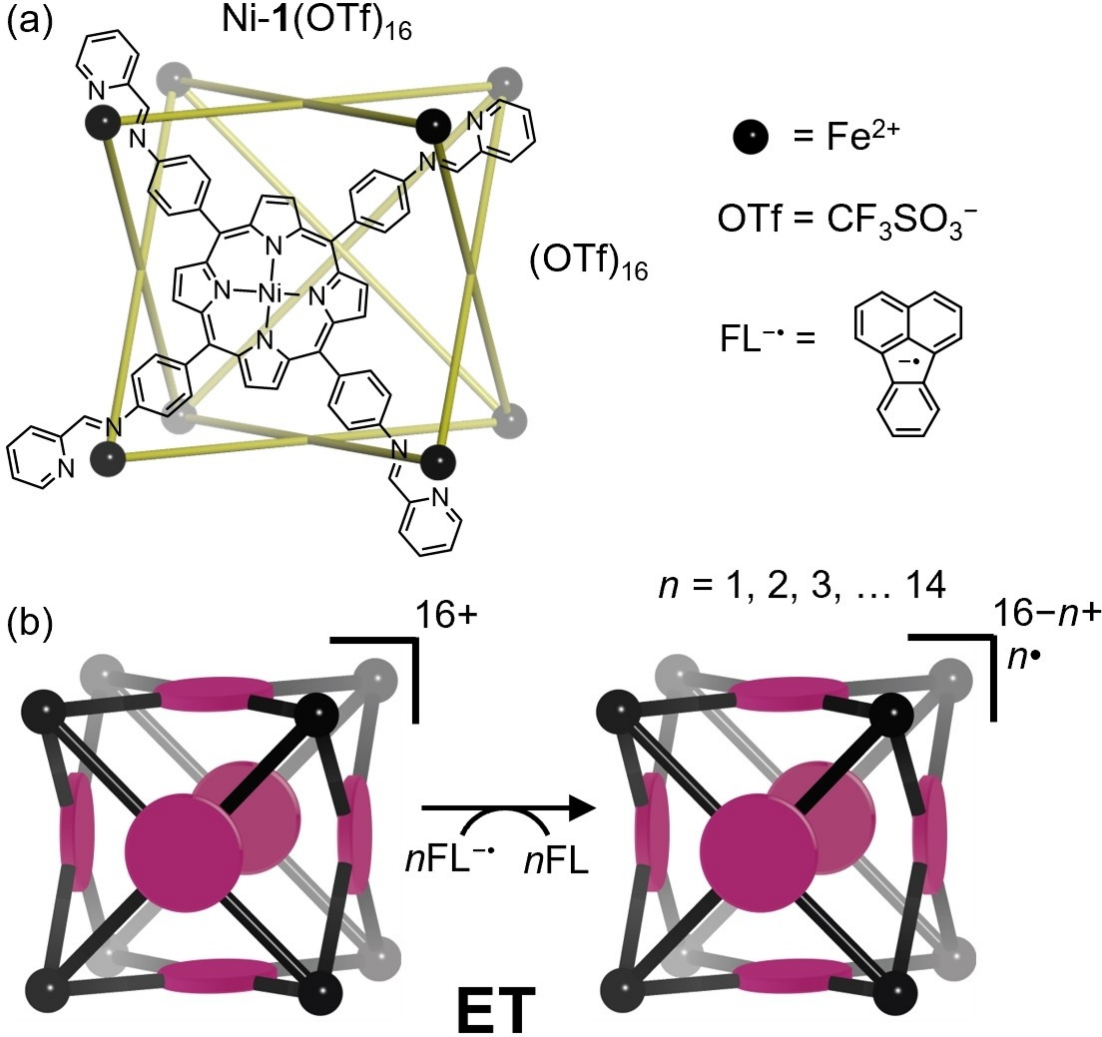
Chemical structure (a) and schematic representation (b) of cage Ni‐**1**(OTf)_16_.

Here we extend this rationale by application to a cationic metalloporphyrin‐faced M_8_L_6_^16+^ coordination cage (Figure 1a), which was reduced to the 2+ oxidation state in 14 steps without significant alteration to the coordination geometry (Figure 1b). The effect of charge state, metalloporphyrin face, and guest encapsulation (C_60_) on the reaction products and rates of each reduction step in the gas phase was explored. The effects of reduction on the reactivity of the metal coordination cages toward molecular O_2_ in the presence and absence of encapsulated C_60_ fullerene were also investigated.

## Results and Discussion

**Figure 2:**
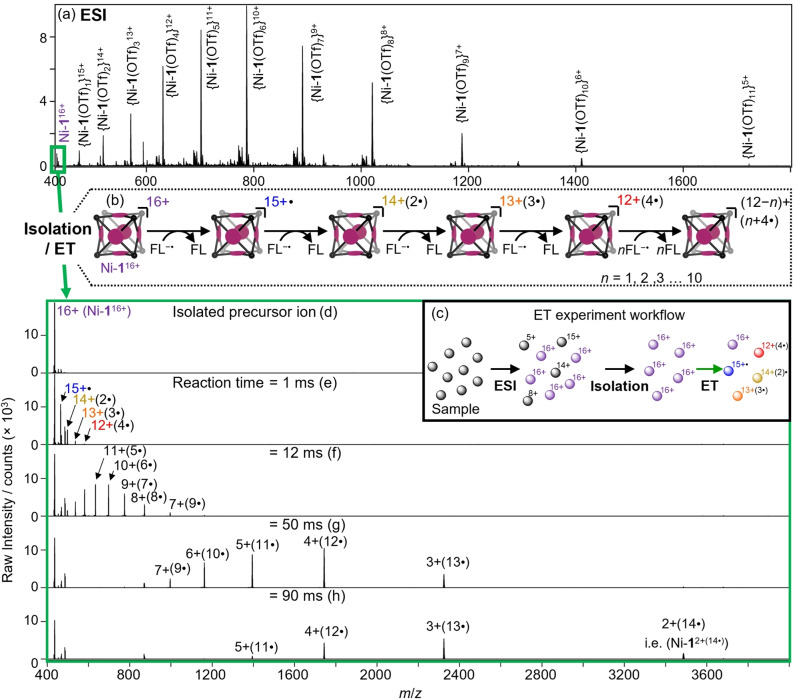
a) ESI mass spectrum of Ni‐**1**(OTf)_16_. b) Scheme depicting gas‐phase sequential electron transfer (ET) reaction of Ni‐**1** ^16+^ with FL^−^⋅. c) Experimental workflow of ET reaction experiments. d) Isolated precursor ion mass spectrum. Mass spectra of the products of the electron transfer (ET) reaction of Ni‐**1** ^16+^ with FL^−^⋅ at various time points (e)–(h) where ⋅ is used to denote the number of added unpaired electrons. Isobaric interferences persisted throughout reaction time windows (Figure S8).

Following a procedure reported by Nitschke et al.,^[17]^ porphyrin‐face capped cubic M_8_L_6_ cage Ni‐**1**(OTf)_16_ (Figure 1a) was prepared by heating an 8 : 6 : 24 mixture of iron(II) triflate, nickel(II) tetrakis(4‐aminophenyl)porphyrin and 2‐pyridinecarboxaldehyde in dimethylformamide until thermodynamic equilibrium was reached (Figure S1, S2). Electrospray ionization (ESI) mass spectrometry of purified Ni‐**1**(OTf)_16_ in acetonitrile yielded a characteristic pattern of positive ions corresponding to the Ni‐**1**(OTf)_(16−*z*)_^*z*+^ (*z*=5–16) cage accompanied by different numbers of residual triflate anions (Figure 2a) along with free cage ions with no associating triflate counter‐ions (i.e., *z*=16^+^_,_ Ni‐**1** ^16+^).

Cations of Ni‐**1** ^16+^ were isolated from the remainder of the array of ESI generated ions (Figure 2a) and stored in the ion trap as a pure population for subsequent experimentation (Figure 2d). In this way, the gas‐phase chemical reactivity of the anion‐free cage could be probed without interference from solvent, matrix, Ni‐**1** cations in different charge states or other extraneous ions. The trapped ions of Ni‐**1** ^16+^ were then reacted with radical anions of fluoranthene (FL^−^⋅, Figure 1), generated from a separate chemical ionization source. After a defined reaction time, the contents of the ion trap were scanned out and the reaction products mass‐analyzed using high‐resolution mass spectrometry (HRMS) (Figure 2c). The ET reaction of FL^−^⋅ with Ni‐**1** ^16+^ for 1 ms resulted in the formation of a series of products with charges ranging from 15^+^ to 12^+^, along with a diminished abundance of the reactant Ni‐**1** ^16+^ (Figure 2e). HRMS revealed that these new charge‐reduced compounds were the products of up to 4 electron transfer reactions from FL^−^⋅ to Ni‐**1** ^16+^(Figure 2b). The reaction between FL^−^⋅ and Ni‐**1** ^16+^ was repeated with reaction times of 12, 50 and 90 ms (Figure 2f–h). Longer reaction times yielded an extension of the series of charge‐reduced products through to the 14‐fold reduced ion Ni‐**1** ^2+(14^⋅^)^ (where ⋅ indicates the number of added electrons). The number of reduction reactions likely exceeds the 14 detectable steps. Observation of the 15^th^‐step product Ni‐**1** ^+(15^⋅^)^ is inhibited by the upper mass‐to‐charge limit of the mass spectrometer while mass spectrometry is unable to detect the neutral product that would be obtained from a 16‐step reduction. Despite the considerable evolution of energy associated with such ion‐ion recombination reactions,^[18]^ no major product ions were observed that could be assigned to dissociation of the coordination network in the precursor or charge‐reduced ions over this reaction time range. That is, the major product ions observed following electrochemical reduction of Ni‐**1** ^16+^ can be assigned to a change in charge rather than a change in molecular mass.

In order to assess the integrity of the cubic structure following reduction, ion‐mobility mass spectrometry (IM‐MS) was employed in tandem with electron transfer. IM‐MS has successfully been applied to the investigation of structural changes in gas‐phase complexes and cages,[^14a^, ^19^] and herein was used to determine the collision cross section of Ni‐1 as it was sequentially reduced. This experiment revealed only a subtle decrease (<6 % change) in gas‐phase collision cross section as Ni‐**1** was reduced over 6 steps from 11^+^ to 5^+^ (Figure S10), indicating that no significant structural rearrangement or degradation occurs. Next the gas‐phase electron transfer experiments were repeated with an identical cage containing a kinetically trapped guest. Guest retention would further indicate that the cage structure remained intact following reduction. To this end, host‐guest complex C_60_⊂Ni‐**1**(OTf)_16_ was prepared according to a literature procedure^[17]^ by the overnight heating of a mixture of Ni‐**1**(OTf)_16_ and fullerene‐C_60_ in deuterated dimethylformamide (Figure S3). ESI of the resulting solution diluted with acetonitrile revealed a new series of ions corresponding to the host‐guest complex C_60_⊂Ni‐**1**(OTf)_(16‐*z*)_^*z*+^, *z*=5–16), consistent with the previously reported data (Figure S6).^[17]^ Using the same workflow as described above, reaction of isolated C_60_⊂Ni‐**1** ^16+^ with FL^−^⋅ over mixing times ranging from 1 to 90 ms yielded charge‐reduced ions corresponding to C_60_⊂Ni‐**1** ^15+^⋅ through to C_60_⊂Ni‐**1** ^2+(14^⋅^)^ (Figure S7). Again, using the electron transfer IM‐MS methodology, no significant structural change or fragmentation was observed throughout the reaction period (Figure S10c, d), nor was there any evidence of free C_60_ or free Ni‐**1**. Together these results confirm that the cage structure is retained throughout each of the 14 reduction steps, thus demonstrating the remarkable stability of these exotic highly reduced species.

**Figure 3:**
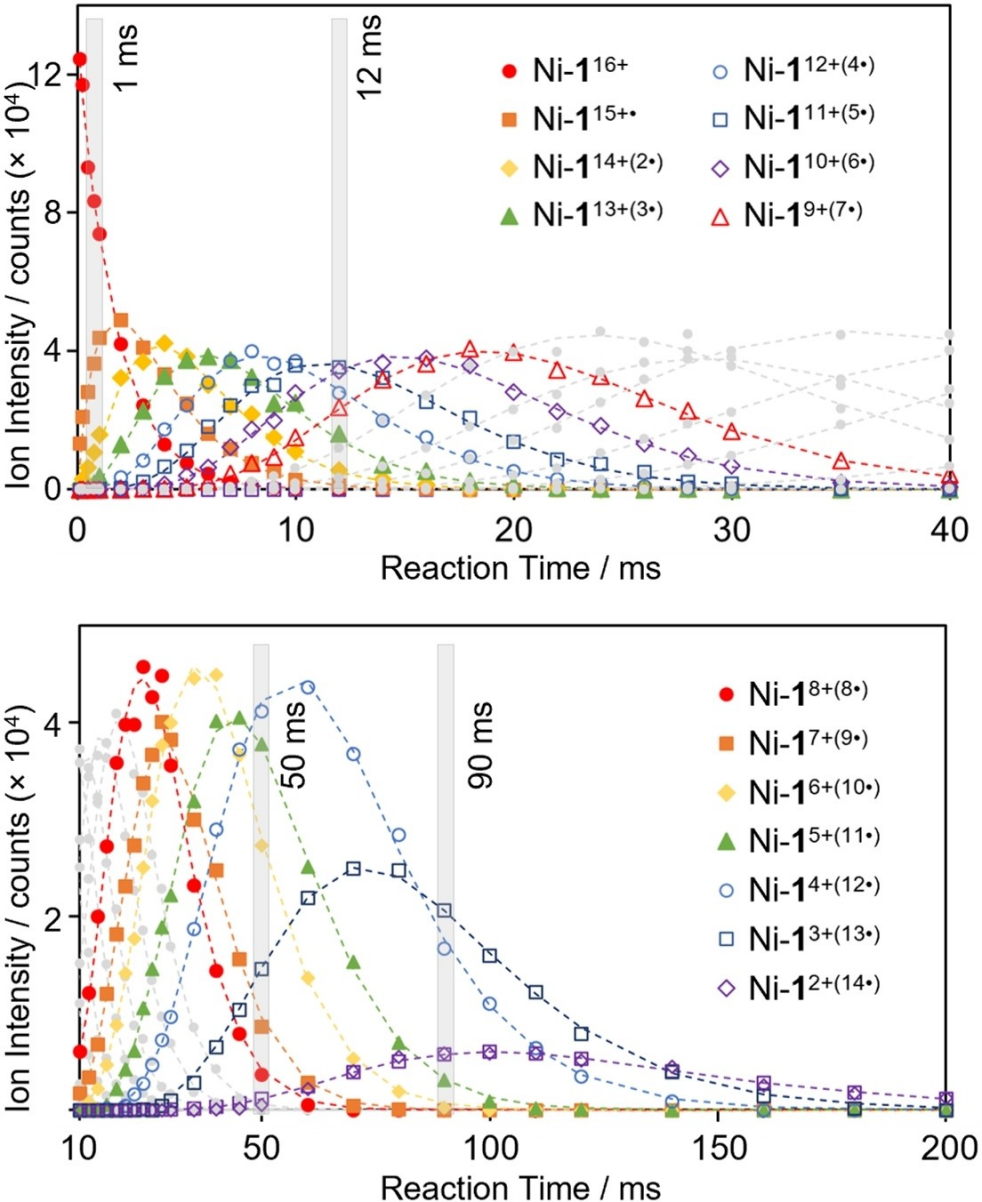
Modelling of the growth and decay kinetics of the products yielded from the reaction of Ni‐**1** ^16+^ with FL^−^⋅ over 200 ms (top 0–40 ms, bottom 10–200 ms) (see Supporting Information for details, model shown using dashed lines). Time domains highlighted in grey pertain to the ET mass spectra shown in Figure 2 (e) to (h).

To gain insights into the reaction pathway leading to the formation of these highly reduced compounds, we set out to model the rate of formation and reaction of each observed species. To this end, the reaction of Ni‐**1** ^16+^ with FL^−^⋅ was monitored in separate experiments ranging from 2 ms to 200 ms. Summed peak intensities of each charge‐reduced species were plotted against time (Figure 3) to graphically visualize their relative growth and decay. The observed transformation of Ni‐**1** ^16+^ to Ni‐**1** ^2+(14^⋅^)^ was modelled as a series of consecutive, irreversible, single electron transfer reactions yielding a rate constant (*k*) for each reduction step (Figure 3). This was accomplished using a general form^[20]^ of the series of solutions of differential equations typically used to determine the rate constants of such ion‐ion reactions,^[21]^ a method which offers the advantage of requiring only a single general equation to model any number of reduction steps (see Supporting Information for details). In order to validate this method, further experiments were carried out wherein each reduced species was explicitly re‐isolated and reacted again with FL^−^⋅. In this way, the reaction rate at each reduction step could also be modelled as a single exponential decay (Table S3). Rate constants obtained through each method were found to be self‐consistent within experimental error. The calculated rate constants were found to decrease linearly with square of the precursor ion charge state (*z* ^2^), (i.e., the reaction slows down as the charge state of the cage ion is reduced) as expected for Coulombic interaction‐driven gas‐phase ion‐ion reactions (Figure S11).^[22]^ The practical effect of this is that product ions resulting from electron transfer are formed faster than they react, giving rise to asymmetrical growth/decay kinetic traces (Figure 3). While these early stages of sequential stepwise electron transfer can be observed in solution (Figure S14),^[23]^ investigations of the properties of the cage at each individual redox state are more accessible in the gas phase where purification through mass‐selection is possible.

These experiments were then repeated with the reaction of FL^−^⋅ with C_60_⊂Ni‐**1** ^16+^ (Figure S12) and the analogous free‐base porphyrin walled cage H_2_‐**1** ^16+^ (Figure S4, S13). Comparison of the corresponding calculated rate constants with those obtained for the reduction of Ni‐**1** ^16+^ (Table 1) revealed that the presence of C_60_ or substitution of the nickel porphyrin for the free base analogue had minimal observable effect on the modelled reaction rates for any of the electron transfer steps. These results suggest that the driving force behind the reduction reactions is predominantly Coulombic in nature, however further investigation into possible influences of chemical structure on reaction rate is ongoing.

**Table 1:** Selected calculated rate constants (*k_i_*) for reactions of FL^−^⋅ with cages in units of s^−1^ (×10^−3^). Absolute uncertainties included in parentheses. Experimental details are provided in the Supporting Information.

*Z*_reactant_→*z*_product_	Ni‐**1**(OTf)_(16−*z*)_^*z*+^	C_60_⊂Ni‐**1**(OTf)_(16−*z*)_^*z*+^	H_2_‐**1**(OTf)_(16−*z*)_^*z*+^
14^+^→13^+^	0.5(1)	0.5(1)	0.5(1)
13^+^→12^+^	0.45(5)	0.47(5)	0.43(4)
12^+^→11^+^	0.36(4)	0.38(4)	0.38(4)
11^+^→10^+^	0.33(3)	0.33(3)	0.34(3)
10^+^→9^+^	0.25(3)	0.28(3)	0.28(3)

**Figure 4:**
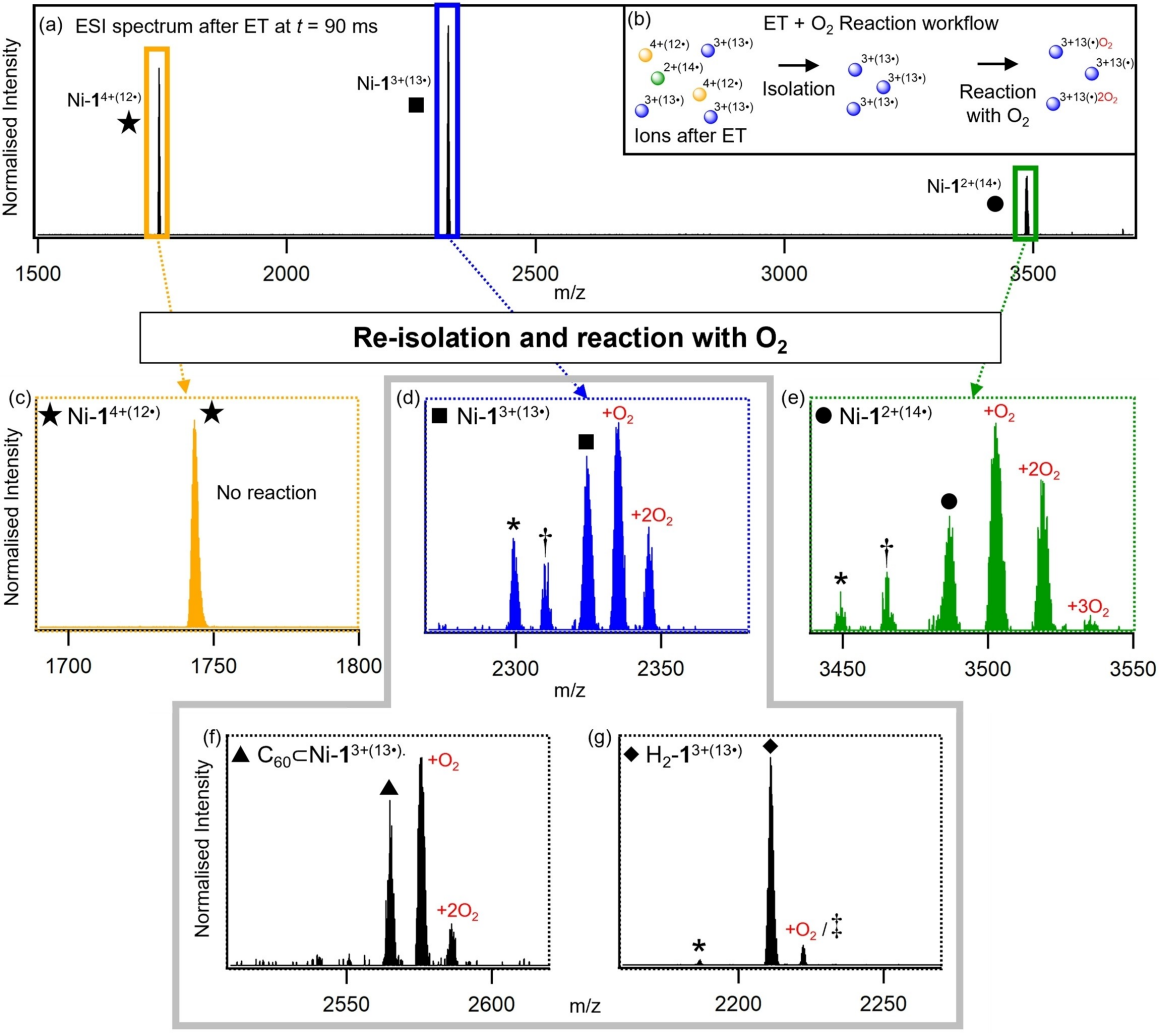
a) ESI‐ET spectrum of Ni‐**1** ^16+^ at *t*=90 ms. b) Workflow for ESI‐ET‐O_2_ reaction methodology. c)–g) Mass spectrum after holding Ni‐**1** ^4+(12^⋅^)^ (c), Ni‐**1** ^3+(13^⋅^)^ (d), Ni‐**1** ^2+(14^⋅^)^ (e), C_60_⊂Ni‐**1** ^3+(13^⋅^)^ (f) and H_2_‐**1** ^3+(13^⋅^)^ (g) in the ion trap with residual O_2_ in the ion trap for 200 ms. Mass to charge of species denoted by * corresponds to [precursor – C_6_H_5_N+O]^*x*+^, † to [precursor – C_6_H_5_N+3 O] and ≠ to [precursor + 2 H_2_O]^3+^ (where *x*=2 (e) or 3 (d,g)).

To probe the chemistry of these highly reduced cage species, their reactivity toward molecular oxygen (O_2_) was investigated. Adventitious background air is always present in the ion trap region of the vacuum system, and thus can be used to study bimolecular reactions of O_2_ with target ions.[^16^, ^24^] This was achieved by first reducing a cage ion of interest to the desired charge state through reaction with FL^−^⋅, followed by re‐isolation of the product ion in the presence of O_2_ (ca. 10^9^ molecules cm^−3^)^[25]^ for a defined period of time (Figure 4b)_._ Reaction products were monitored in situ via high‐resolution mass spectrometry.

Using this approach, significant variation in reactivity of cage ions toward O_2_ was observed as a function of charge state, number of added electrons, the presence of C_60_ guest and substitution of the porphyrin faces. Much of this variation was evident upon examination of the reactivity of Ni‐**1** ^16+^ and its reduced forms (Figure 4c–e), along with comparison with the C_60_ containing and free‐base porphyrin analogues (Figure 4f, g respectively). First, ions of Ni‐**1** ^16+^ were isolated and held in the ion trap for reaction with O_2_ without any prior reduction steps. No reaction was observed over the period of 2000 ms. Following this, each sequentially reduced ion was separately isolated in the presence of residual O_2_ and the reaction products monitored over 2000 ms. While all reduced species up to and including Ni‐**1** ^4+(12^⋅^)^ were observed to be inert towards O_2_ over this time range (Figure 4c), holding reduced species Ni‐**1** ^3+(13^⋅^)^ in the ion trap for just 200 ms produced a significant quantity of various O_2_ adducts (Figure 4d, Figure S15). Among these, additions of either one or more O_2_ were observed as well as losses of a methinyl pyridine group accompanied by the addition of a single oxygen atom, implicating the pyridyl‐imine groups coordinated to Fe to be sites of O_2_ reactivity. The proportion of these products further increased with reduction of the precursor ion to Ni‐**1** ^2+(14^⋅^)^ (Figure 4e). Intriguingly, the threshold number of added electrons required for this reactivity to be “switched‐on” is equal to twice the number of Ni porphyrin units (Ni(P)) plus one. Taking into account the well‐known capacity of nickelated porphyrins to accept two electrons,^[26]^ we propose that the first 12 reductions of Ni‐**1** ^16+^ result in two electrons added to each Ni(P), with subsequent further reduction steps having the potential to yield radical sites (located on the pyridyl‐imine groups), which readily react with trace molecular oxygen to give rise to the observed loss of methinyl pyridine. These results, coupled with the lack of O_2_ reactivity of the equivalently charged but un‐reduced species Ni‐**1**(OTf)_13_^3+^, suggest that the presence of additional electrons plays an important role in the observed reactivity. Given the requirement for 13 reduction steps before reactivity is observed, access to this chemistry is likely not possible within the normal realms of redox states available to solution or solid‐state studies.

Identical reactivity screening was then performed on the C_60_‐containing and free‐base porphyrin analogues C_60_⊂Ni‐**1** ^3+(13^⋅^)^ and H_2_‐**1** ^3+(13^⋅^)^. Incorporation of a guest C_60_ within the Ni‐**1** cage significantly influenced its reactivity with O_2_. While 13 reduction steps were still necessary to “switch‐on” reactivity, the reaction of C_60_⊂Ni‐**1** ^3+(13^⋅^)^ with O_2_ was significantly retarded compared with empty host analogue Ni‐**1** ^3+(13^⋅^)^ after 200 ms (Figure 4f), suggesting that encapsulated C_60_ serves as an electron sink in the former. Interestingly, only very minor evidence of reaction with O_2_ was observed for H_2_‐**1** ^3+(13^⋅^)^. These findings suggest the possibility of significant tunability in the chemistry of these highly reduced supramolecular species, accessible through variations in controllable structural parameters, such as the identities of the metals, porphyrin ligands and guests.

## Conclusion

Three metallosupramolecular cages were sequentially reduced up to 14 times in the gas phase, without compromising structural integrity. This remarkable result highlights the structural stability of these cages in highly reduced forms accommodating up to 14 extra electrons. The rate constants at each reduction step were determined using a general form of previously used equations allowing for more streamlined modelling of multiple reaction steps. Although neither C_60_ guest inclusion nor changes to cage chemical structure affected the observed rate constants, both had significant implications on reactivity toward O_2_. The reactivity of these reduced supramolecular species can therefore be tuned by varying well‐controlled structural parameters, which could be made to include the incorporation of molecular components with specific functionalities, such as catalysis. We anticipate that this technique will have broad applicability for the investigation of the interesting redox activity and chemistry of cationic supramolecular complexes and the intrinsic chemical nature of highly reduced supramolecular species, without complications arising from solvents, impurities or degradation.

## Conflict of interest

The authors declare no conflict of interest.

1

## Supporting information

As a service to our authors and readers, this journal provides supporting information supplied by the authors. Such materials are peer reviewed and may be re‐organized for online delivery, but are not copy‐edited or typeset. Technical support issues arising from supporting information (other than missing files) should be addressed to the authors.

Supporting Information

## Data Availability

The data that support the findings of this study are available in the Supporting Information of this article.
