# Supplementary material for: Exploring the Gas‐Phase Formation and Chemical Reactivity of Highly Reduced M8L6 Coordination Cages
Source: Angew Chem Int Ed Engl. 2022 Oct 7;61(45):e202212710. doi: 10.1002/anie.202212710 (PMC9827999; doi:10.1002/anie.202212710)
Supplement: Supplementary file 1 — Supporting Information [file ANIE-61-0-s001.pdf]

Angewandte Chemie
Eine Zeitschrift der Gesellschaft Deutscher Chemiker

Supporting Information

Exploring the Gas-Phase Formation and Chemical Reactivity of Highly Reduced M_8L_6 Coordination Cages

*M. C. Pfrunder, D. L. Marshall, B. L. J. Poad, E. G. Stovell, B. I. Loomans, J. P. Blinco, S. J. Blanksby, J. C. McMurtrie, K. M. Mullen**

SUPPORTING INFORMATION

Exploring the Gas-Phase Formation and Chemical Reactivity of Highly Reduced M_8L_6 Coordination Cages

Michael C. Pfrunder,[a,b] David L. Marshall,[a,c] Berwyck L. J. Poad,[a-c] Ethan G. Stovell,[a] Benjamin I. Loomans,[a,b] James P. Blinco,[a,b] Stephen J. Blanksby,[a-c] John C. McMurtrie,[a,b] and Kathleen M. Mullen[a,b]*

[a] Centre for Materials Science, Queensland University of Technology (QUT), 2 George Street, Brisbane, Queensland 4000, Australia

[b] School of Chemistry and Physics, QUT, 2 George Street, Brisbane, Queensland 4000, Australia

[c] Central Analytical Research Facility (CARF), Institute for Future Environments (IFE), QUT, 2 George Street, Brisbane, Queensland 4000, Australia

*Corresponding Author

Table of Contents

Synthesis and Characterisation 2

Electron Transfer Mass Spectrometry 5

Ion Mobility Mass Spectrometry 7

Electron Transfer Reaction Kinetics 10

Cyclic Voltammetry 15

Dioxygen Reactivity Investigations 16

Comparisons of Experimental Mass Spectrometry Data to Theoretical Simulations 17

References 23

Synthesis and Characterisation

General Materials and Considerations

Tetrakis(4-aminophenyl)porphyrin was obtained from Combiblocks and 2-pyridinecarboxaldehyde and C_{60}-fullurene from Merck Sigma-Aldrich and used as received. Tetrakis(4-aminophenyl)porphyrin nickel(II) and cages Ni-**1**, C_{60}⊂Ni-**1** and H_2-**1** were prepared according to literature procedures.[1] Solution nuclear magnetic resonance (NMR) spectra were recorded on either a Bruker Avance 600 or 400 MHz spectrometer and referenced to the relevant solvent peak. When mixtures of DMF-d_7 and acetonitrile-d_3 were used, spectra were referenced to the residual solvent peak of the latter.

Synthesis of *Tetrakis(4-aminophenyl)porphyrin nickel(II)*

A 1:3 dimethylformamide (DMF) / chloroform mixture (40 cm^3) was sparged with Ar for 10 minutes before precursor tetrakis(4-aminophenyl)porphyrin (51.5 mg, 76.3 µmol) was added and dissolved. Nickel(II) acetate tetrahydrate (76.0 mg, 305 µmol) was suspended in methanol (5 cm^3) and sonicated for 10 minutes and the resulting cloudy solution was added to the precursor mixture. The head space of the flask was purged with Ar and the reaction mixture was heated at reflux for 24 hours. The resulting red solution was extracted with water (5 × 15 cm^3) and the precipitating dark purple solid, tetrakis(4-aminophenyl)porphyrin nickel(II), was collected via vacuum filtration (39.6 mg, 71 %). ^{1}HNMR (DMSO-d6, 600 MHz): 8.77 (s, 8H), 7.63 (d, 8H, 8.3 Hz), 6.92 (d, 8H, 8.3 Hz), 5.50 (s, 8H).

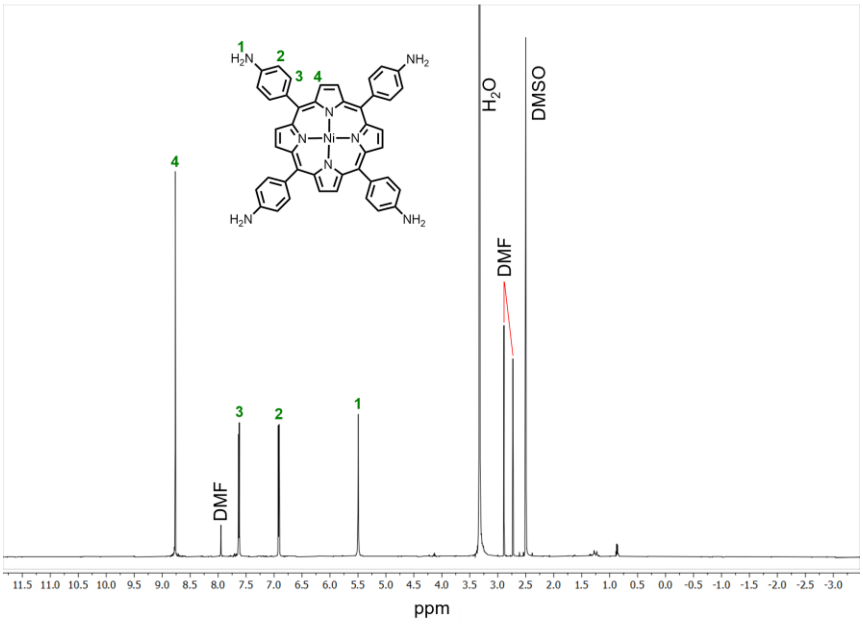

Figure S1: ^{1}H NMR spectrum of tetrakis(4-aminophenyl)porphyrin nickel(II) in dimethylsulfoxide-d6 (DMSO-d6).

*Synthesis of Ni-**1***

Tetrakis(4-aminophenyl)porphyrin nickel(II) (19.6 mg, 26.8 µmol, 6 eq.) was dissolved in dry, Ar-sparged DMF (0.4 cm^3). With stirring, a suspension of iron(II) triflate (12.6 mg, 35.6 µmol, 8 eq.) was added with a further 0.3 cm^3 of DMF. To this was added 2-pyridylcarboxaldehyde (10.24 µL, 11.53 mg, 107.6 µmol, 24 eq.) and the reaction mixture was sparged with Ar for 5 minutes and heated at 70 °

C under Ar overnight. The mixture was allowed to cool and diethyl ether (10 cm^3) was added. The resulting purple precipitate was filtered onto celite, washed with a further 10 cm^3 of diethyl ether and dissolved and washed through the celite with freshly sparged DMF. The DMF was removed under a stream of N_2 gas overnight to give Ni-**1** as a fine purple powder (26.8 mg, 75 %) ^{1}HNMR (acetonitrile-d3/DMF-d7 3:2, 600 MHz): 9.72 (s, 24H), 9.20 (br, 24H), 8.84 (m, 48H), 8.53 (br, 24H), 8.07 (d, overlapped with solvent), 7.75 (d, 24H, 5.5 Hz), 7.50 (br, 24H), 6.30 (br, 24H), 5.80 (br, 24H). Selected ions from ESI-MS: Ni-$\mathbf{1}^{16+}$ 435.84, Ni-**1**$(OTf)_3^{13+}$ 570.79, Ni-**1**$(OTf)_9^{7+}$ 1187.85.

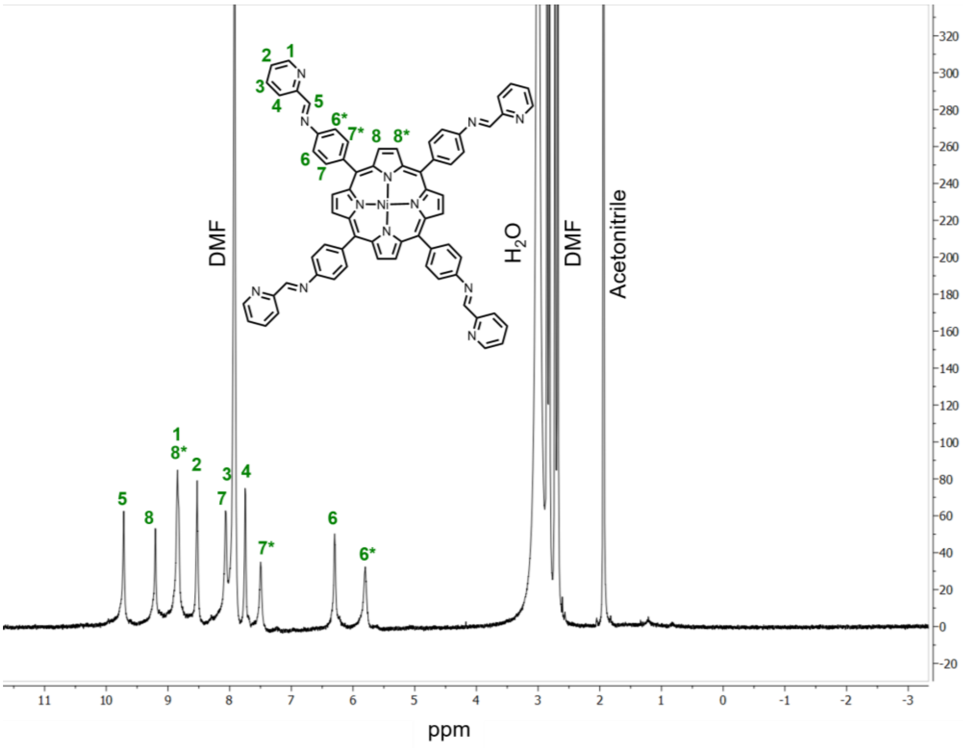

Figure S2: ^{1}H NMR spectrum of Ni-**1** in 3:2 acetronitrile-d3 / DMF-d7.

*Synthesis of C_{60}⊂Ni-**1***

Cage Ni-**1** (3.8 mg, 0.48 µmol) and fullerene C_{60} (2.0 mg, 2.8 µmol) were taken up in 0.4 cm^3 of Ar sparged DMF-d7 in an NMR tube. The atmosphere was purged with Ar for 2 minutes and the tube was sealed and heated at 70 º C overnight. Selected ions from ESI-MS: C_{60}⊂Ni-**1**$(OTf)_3^{13+}$ 626.17, C_{60}⊂Ni-**1**$(OTf)_5^{11+}$ 767.11, C_{60}⊂Ni-**1**$(OTf)_9^{7+}$ 1290.71.

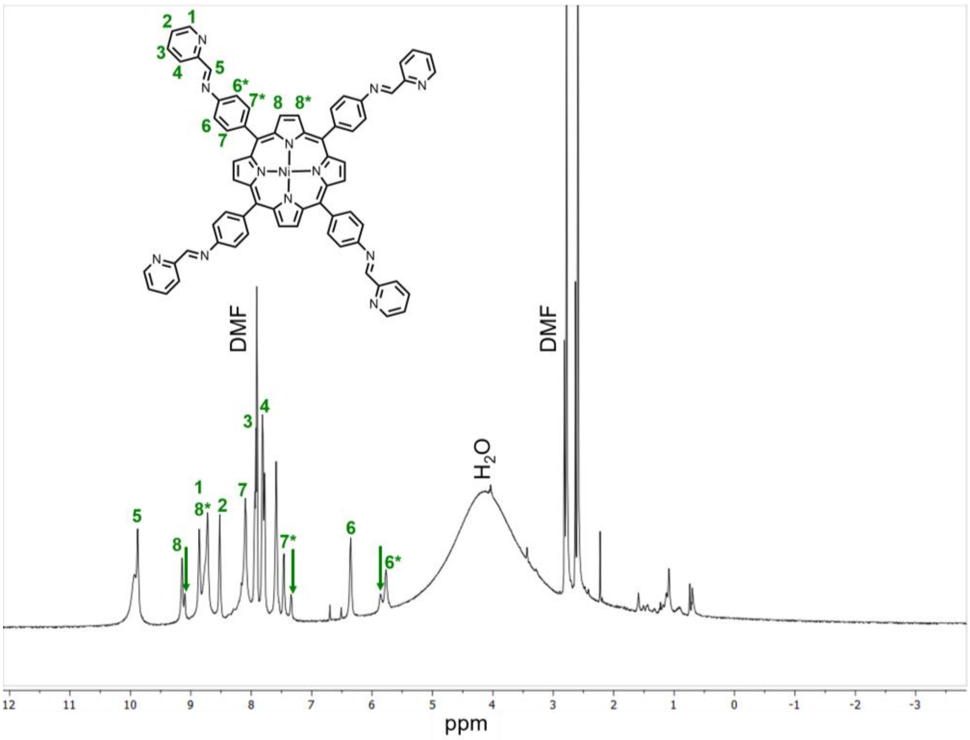

Figure S3: ^{1}H NMR spectrum of C_{60}⊂Ni-**1** in DMF-d7. Resonances pertaining to guest encapsulated species indicated with green arrows.[1]

*Synthesis of H_2-**1***

Tetrakis(4-aminophenyl)porphyrin (20.2 mg, 29.9 µmol, 6 eq.) was dissolved in dry, Ar-sparged DMF (0.7 cm^3). With stirring, a suspension of iron(II) triflate (14.1 mg, 39.9 µmol, 8 eq.) was added with a further 0.7 cm^3 of DMF. To this was added 2-pyridylcarboxaldehyde (11.40 µL, 12,83 mg, 119.7 µmol, 24 eq.) and the reaction mixture was sparged with Ar for 5 minutes and heated at 70 º C under Ar overnight. The mixture was allowed to cool and diethyl ether (10 cm^3) was added. The resulting purple precipitate was filtered onto celite, washed with a further 10 cm^3 of diethyl ether and dissolved and washed through the celite with freshly sparged DMF. The DMF was removed under a stream of N_2 gas overnight to give H_2-**1** as a fine purple powder (21.8 mg, 81 %) ^{1}HNMR (^{1}HNMR (acetonitrile-d3/DMF-d7 3:2, 600 MHz): 9.66 (s, 24H), 9.30 (s, 24H), 8.93 (s, 24H), 8.83 (br, 24H), 8.52 (t, 24H, 6.5 Hz), 8.16 (br, 24H), 7.91 (overlapped with residual DMF signal) 7.71 (d, 24H, 5.0 Hz, 7.61 (br, 24H), 6.33 (br, 24H), 5.85 (br, 24H), −3.78 (br, 12H). Selected ions from ESI-MS: H_2-**1**$(OTf)_3^{13+}$ 544.67, H_2-**1**$(OTf)_5^{11+}$ 670.78, H_2-**1**$(OTf)_7^{9+}$ 852.95.

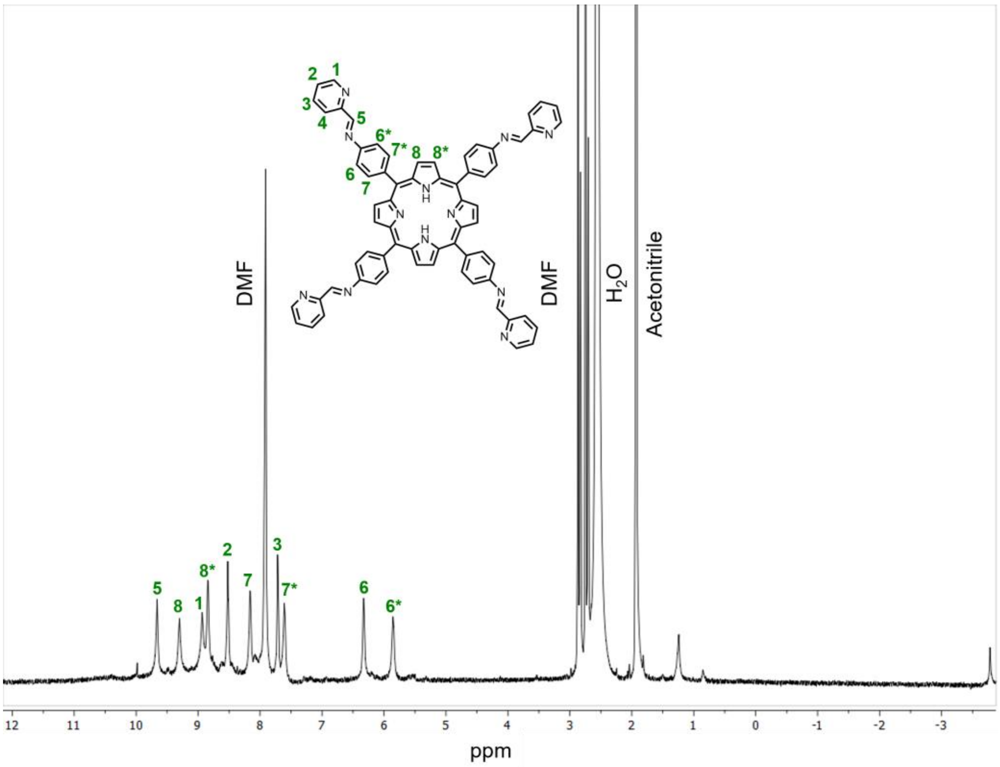

Figure S4: ^{1}H NMR spectrum of H_2-**1** in 3:2 acetronitrile-d3 / DMF-d7.

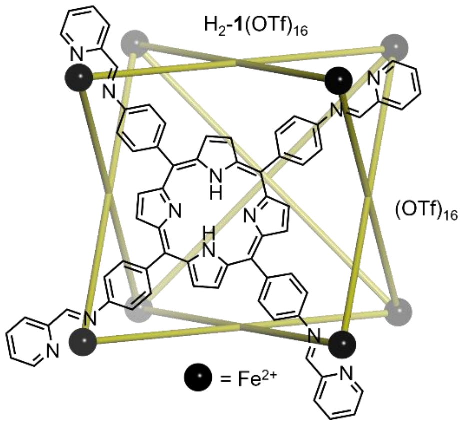

Figure S5: Structure of cage H_2-**1**$(OTf)_{16}$.

Electron Transfer Mass Spectrometry

All experiments were performed with a high-resolution Orbitrap Elite mass spectrometer (Thermo Fisher Scientific, Bremen, Germany) operating at a mass resolution of 60,000 (at *m*/*z* 400). Mass calibration in positive ion mode was performed prior to use. Target compounds were dissolved in acetonitrile at approximately 0.1 mg/mL, then diluted further into acetonitrile to achieve a final concentration of ca. 0.5 µM. Diluted samples were infused into the heated electrospray ionisation (HESI) source at 15 µL/min. Typical ion source parameters were source voltage 4.2 kV, capillary temperature 275 °C, sheath gas 25 arbitrary units, auxiliary gas 2 arbitrary units, S-lens RF level 53%. Mass-to-charge ratios of precursor ions and reaction products were within 5 ppm of their predicted values (Tables S4-9), using the most abundant peak in the isotopic distribution (base peak) for

comparison. Ion-ion reactions of cage cations with fluoranthene radical anions ($FL^{-\bullet}$) were performed using the electron transfer dissociation (ETD) module of the Orbitrap Elite,[2] with a reagent vial temperature of 108 °C and an automatic gain control (AGC) target set to 4 × 10^5. Multiply charged analyte cations (from ESI) were mass-selected in the ion trap with the smallest isolation window allowing maximum retention of ions of interest (2.5 - 15 Da) and the ion trap stability parameter (*q*) set to 0.25. Fluoranthene ions were generated by chemical ionisation and transferred into the ion trap for a given reaction time before the cationic reaction products were scanned out and analysed in the Orbitrap.

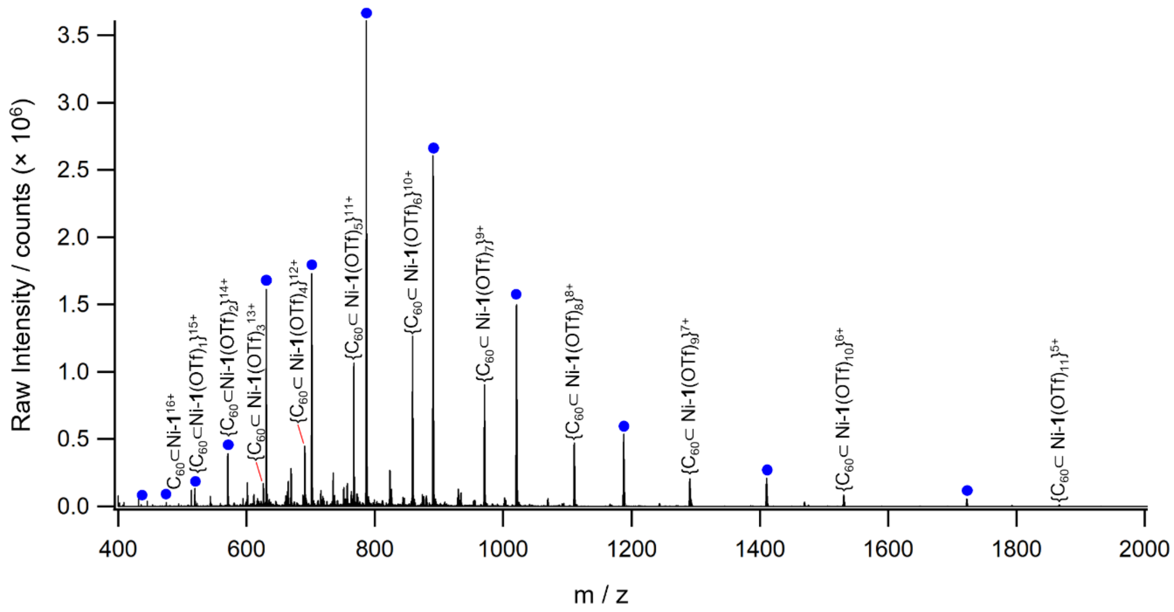

Figure S6: ESI spectrum of C_{60}⊂Ni-**1**. Peaks corresponding to empty host Ni-**1** species denoted with blue circles.

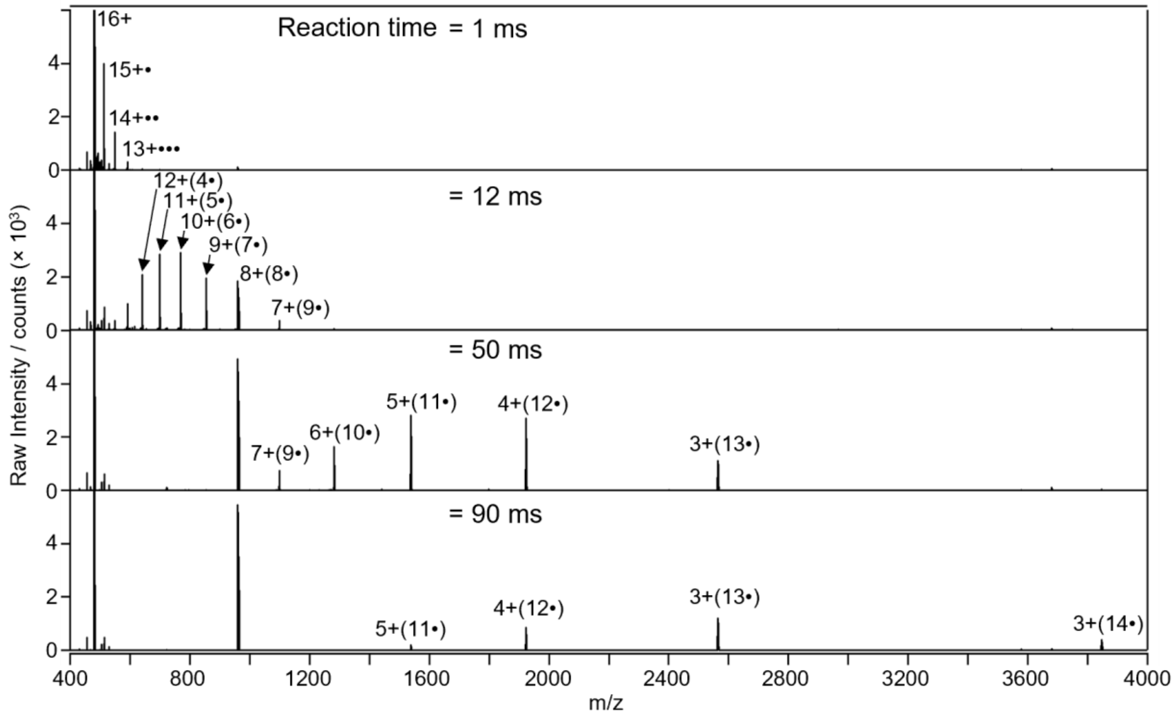

Figure S7: Mass spectra of the products of the electron transfer (ET) reaction of C_{60}⊂Ni-**1**$^{16+}$ with $FL^{-\bullet}$ at various time points.

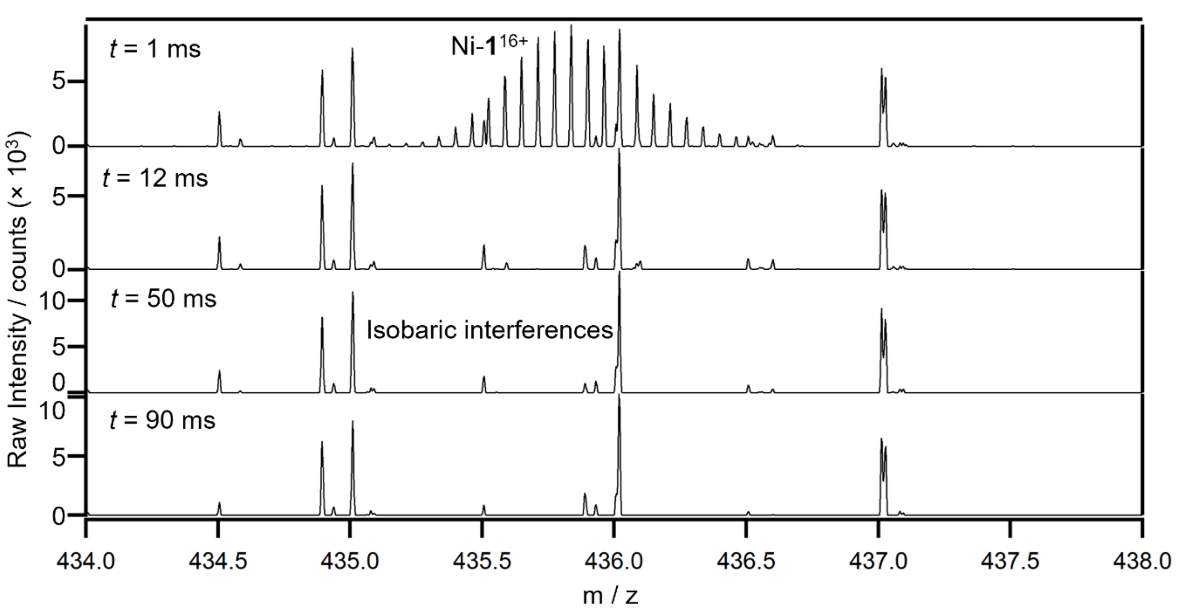

Figure S8: ESI-ET spectra showing the rapid decay of Ni-**1**$^{16+}$ between 1 and 12 ms while isobaric interferences remain. Isolation window size set to the smallest range possible that allowed maximum retention of ions of interest (2.5 Da).

Ion Mobility Mass Spectrometry

Travelling wave ion mobility mass spectrometry (TWIM-MS) uses a travelling electric field to transmit ions through a buffer gas.[3] The time taken for ions to complete this transit is known as drift time. Following instrument calibration using known standards, drift time can be used to determine an ion's collision cross section (CCS), which is related to the ion's size and shape. Changes in CCS occurring over the course of a series of chemical reactions can inform on the presence of resulting conformational rearrangements.[4]

TWIM-MS experiments of gas phase electrochemically reduced ions were performed on a Waters Synapt G2-S*i* mass spectrometer in "ETD" mode with 1,4-dicyanobenzene radical anions (*m/z* 127) generated from a glow-discharge source as the gas phase electron donor. The spray capillary voltage, sampling cone and source offset were set to 2.5 kV, 0 V and 0 V respectively. The trap entrance, bias, trap DC and exit were set at −3, 30, −3 and 10 V. Mobility separation experiments were carried out on electrochemically reduced ions that had been quadrupole mass-selected using settings adapted from a previous report[4] with a travelling wave velocity of 1500 ms^{-1} and wave height of 40 V in the ion mobility cell, and 220 ms^{-1} and 4 V in the transfer cell. Trap and transfer cells were filled with Ar at 2.4×10^{-2} and 2.8×10^{-2} mbar respectively, the helium cell with He at 3.81 mbar and the ion mobility with N_2 at a pressure of 2.98 mbar. Radical anions were generated with a glow discharge current of 55 µA and a make-up gas flow of 30 $mLmin^{-1}$. The ion mobility cell was calibrated for N_2 collisional cross section (CCS) determination according to a previously described method[5] using the arrival times of cytochrome C (equine) and Myoglobin (equine) dissolved in 50:50 methanol:water. Drift tube IM-MS nitrogen buffer gas CCS reference values were obtained from a previous report.[6]

In individual experiments, mass-selected ions of Ni-**1**$(OTf)_5^{11+}$, Ni-**1**$(OTf)_7^{9+}$, C_{60}⊂Ni-**1**$(OTf)_5^{11+}$ and C_{60}⊂Ni-**1**$(OTf)_7^{9+}$ were subjected to gas phase radical ions and the resulting electrochemically reduced product ions were analysed using TWIM-MS (Figure S9 a to d respectively). It was found that only small contractions in CCS occurred as ions were reduced from 11+ to 5+ ($\Delta\ {}^{TW}CCS_{N2}$ *ca.* 5 %, Figure S10 and Table S1). By comparison to previously reported changes in CCS values associated with the

electrochemical reduction induced molecular rearrangement of a foldamer over the same range of charge states (Δ CCS *ca*. 30%),[4] the small CCS changes observed here indicate that only subtle conformational changes occur during the reduction of the cages reported herein.

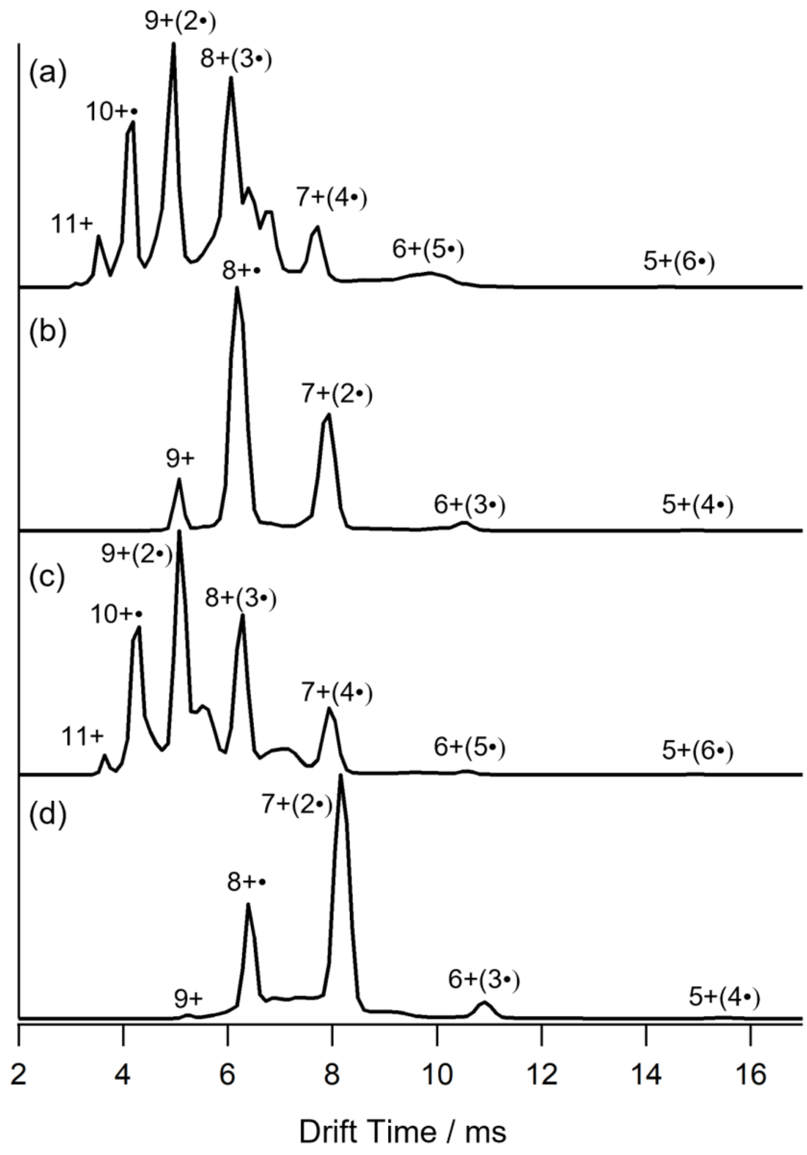

Figure S9: TWIM-MS mobilograms of precursor ions (a) Ni-**1**$(OTf)_5^{11+}$, (b) Ni-**1**$(OTf)_7^{9+}$, (c) $C_{60}\subset$Ni-**1**$(OTf)_5^{11+}$ and (d) $C_{60}\subset$Ni-**1**$(OTf)_7^{9+}$ along with corresponding sequentially reduced ions.

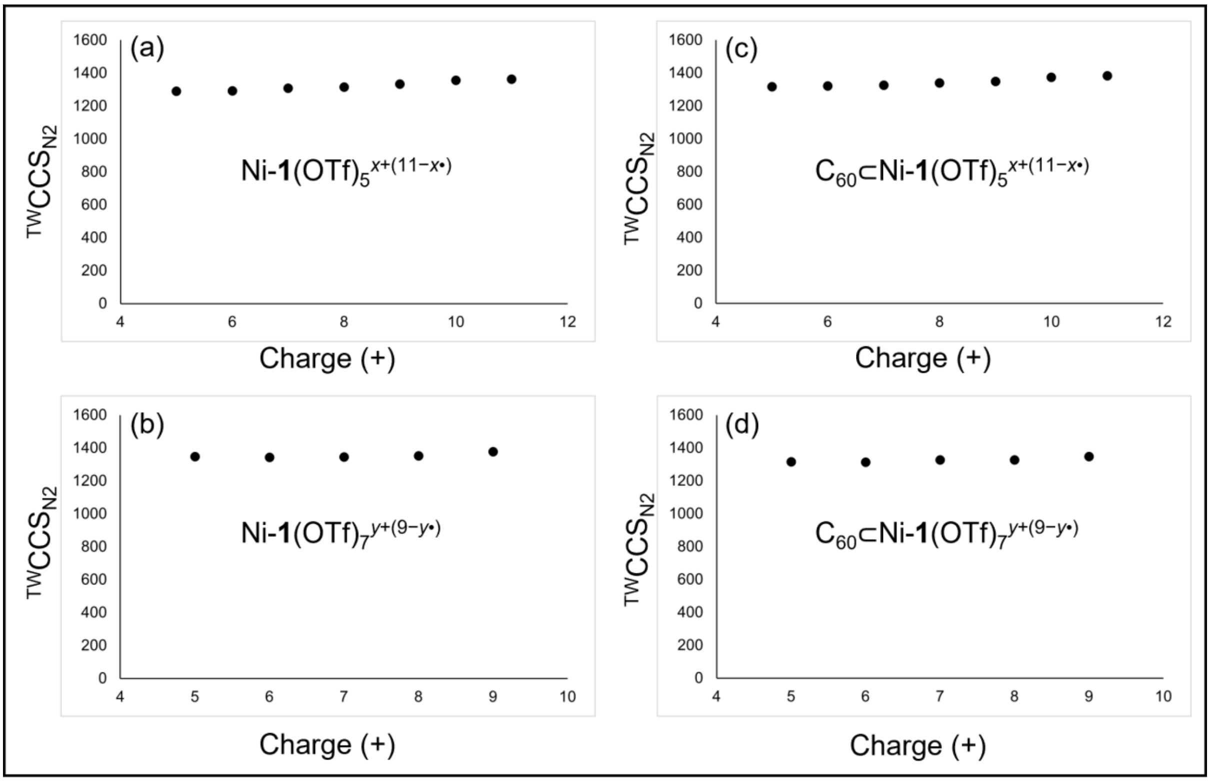

Figure S10: Plots depicting observed changes in ion CCS as cage ions undergo sequential gas phase electrochemical reduction, where *x* = 11 to 6 (a, c) and to *y* = 9 to 5 (b, d).

Table S1: Nitrogen buffer gas CCS values of species calculated from TWIM-MS experiments shown in in Figure S9.

**Assignment**	$^{TW}CCS_{N2}$ (Å^2)	**Assignment**	$^{TW}CCS_{N2}$ (Å^2)
Ni-**1**(OTf)$_5^{11+}$	1362.15	$C_{60}\subset$Ni-**1**(OTf)$_5^{11+}$	1384.00
Ni-**1**(OTf)$_5^{10+\bullet}$	1355.02	$C_{60}\subset$Ni-**1**(OTf)$_5^{10+\bullet}$	1373.32
Ni-**1**(OTf)$_5^{9+(2\bullet)}$	1332.44	$C_{60}\subset$Ni-**1**(OTf)$_5^{9+(2\bullet)}$	1347.62
Ni-**1**(OTf)$_5^{8+(3\bullet)}$	1315.61	$C_{60}\subset$Ni-**1**(OTf)$_5^{8+(3\bullet)}$	1340.25
Ni-**1**(OTf)$_5^{7+(4\bullet)}$	1306.93	$C_{60}\subset$Ni-**1**(OTf)$_5^{7+(4\bullet)}$	1326.15
Ni-**1**(OTf)$_5^{6+(5\bullet)}$	1292.21	$C_{60}\subset$Ni-**1**(OTf)$_5^{6+(5\bullet)}$	1321.14
Ni-**1**(OTf)$_5^{5+(6\bullet)}$	1290.53	$C_{60}\subset$Ni-**1**(OTf)$_5^{5+(6\bullet)}$	1316.10
Δ $^{TW}CCS_{N2}$ Ni-**1**(OTf)$_5^{11+}$ → Ni-**1**(OTf)$_5^{5+(6\bullet)}$	−5.6 %	Δ $^{TW}CCS_{N2}$ $C_{60}\subset$Ni-**1**(OTf)$_5^{11+}$ → $C_{60}\subset$Ni-**1**(OTf)$_5^{5+(6\bullet)}$	−5.2 %
Ni-**1**(OTf)$_7^{9+}$	1347.78	$C_{60}\subset$Ni-**1**(OTf)$_7^{9+}$	1377.97
Ni-**1**(OTf)$_7^{8+\bullet}$	1328.00	$C_{60}\subset$Ni-**1**(OTf)$_7^{8+\bullet}$	1352.45
Ni-**1**(OTf)$_7^{7+(2\bullet)}$	1326.27	$C_{60}\subset$Ni-**1**(OTf)$_7^{7+(2\bullet)}$	1345.23
Ni-**1**(OTf)$_7^{6+(3\bullet)}$	1314.01	$C_{60}\subset$Ni-**1**(OTf)$_7^{6+(3\bullet)}$	1342.52
Ni-**1**(OTf)$_7^{5+(4\bullet)}$	1316.18	$C_{60}\subset$Ni-**1**(OTf)$_7^{5+(4\bullet)}$	1346.76
Δ $^{TW}CCS_{N2}$ Ni-**1**(OTf)$_7^{9+}$ → Ni-**1**(OTf)$_7^{5+(4\bullet)}$	−2.4 %	Δ $^{TW}CCS_{N2}$ $C_{60}\subset$Ni-**1**(OTf)$_7^{9+}$ → $C_{60}\subset$Ni-**1**(OTf)$_7^{5+(4\bullet)}$	−2.3 %

Electron Transfer Reaction Kinetics

Ion-ion reaction kinetics in an ion trap have been detailed by McLuckey and co-workers.[7] Primarily, the reaction rate is proportional to the square of the number of charges.[7b] As long as the local concentration of $FL^{-•}$ greatly exceeds that of the cation and q is held constant, the stepwise electrochemical reduction of multiply-charged cage cations can be described by pseudo-first order kinetics of consecutive reactions.[8] To confirm pseudo-first order conditions, rate constants were measured at different $FL^{-•}$ number densities by adjusting the automatic gain control. Above a critical AGC threshold (4×10^5), the obtained rate constants were found to be independent of the $FL^{-•}$ number density.[8b, 9] The reaction time was varied from 1 – 200 ms in random order to account for temporal fluctuations in fluoranthene ion concentration.

The reaction of a cage ion in the z+ charge state (C^{z+}) with $FL^{-•}$ forms a radical cation $C^{(z-1)+•}$ and neutral FL (Equation 1). Further reduction with a second $FL^{-•}$ yields $C^{(z-2)+••}$ and so on. Rate constants were obtained using changes in the raw intensities of the precursor ion and each charge-reduced product ion as a function of reaction time. Schmidt and co-workers have described a method for estimating the rate constants of consecutive irreversible reactions.[10] First, k_1 was obtained by fitting the decay of C^{z+} to a single exponential equation (Equation 2). Next, the abundance of $C^{(z-1)+}$ was plotted against reaction time and fitted to Equation 3[10] with the least squares method using k_1 from Equation 2, yielding k_2. The rate constants $k_3 \ldots k_n$ were obtained sequentially in the same way, with each subsequent species fit using the rate constants calculated for the preceding reaction steps. The first term, $\sum_{j=1}^{i} x_j(0)$ was also refined during each step using the least squares method, correcting for differences in instrument mass sensitivity across the entire m/z range. Typically, the entire isotopic cluster would be integrated to calculate the ion abundance at each reaction time point, however when significant isobaric interference was present around the precursor ion as a consequence of imperfect ion isolation, as in the case for Ni-**1**$^{16+}$ (Figure S3), only a single, well-resolved isotope peak was used for all ions in the data set.

$$C^{z+} + FL^{-•} \xrightarrow{k_1} C^{(z-1)+•} + FL \qquad \text{(Equation 1)}$$

$$C^{(z-1)+•} + FL^{-•} \xrightarrow{k_2} C^{(z-2)+••} + FL$$

$$C^{(z-2)+••} + FL^{-•} \xrightarrow{k_3} C^{(z-3)+•••} + FL$$

$$C^{(z-(n-1))+(n-1•)} + FL^{-•} \xrightarrow{k_n} C^{(z-n)+(n•)} + FL$$

$$C^{z+}(t) = C_0^{z+} e^{-k_1 t} \qquad \text{(Equation 2)}$$

$$C_i(t) = \sum_{j=1}^{i} C_j(0) \left(\prod_{r=j}^{i-1} k_r \right) \sum_{r=j}^{i} \frac{\exp(-k_r t)}{\prod_{m=j, \, \neq r}^{i} (k_m - k_r)}, \qquad \text{(Equation 3)}$$

where $\quad k_i \neq k_j \quad$ for $\quad i \neq j,$

C_i is the abundance of the ith species and all undefined products are 1.

The reproducibility of rate constants determined using this method was assessed by comparing the results of a series of experiments repeated 7 times over a period of 10 days. Precursor ions for this study were selected based on which were obtained in the greatest abundance from the ESI (Figure S6). The experiments involved the $FL^{-•}$ induced electrochemical reduction of precursor ions of Ni-**1**$(OTf)_5^{z+}$ (x = 2 to 11), or C_{60}⊂Ni-**1**$(OTf)_5^{y+}$ (y = 3 to 11) (Table S2). Using Equation 3, rate constants for each reduction step from each replicate were calculated and from these a mean rate constant for each reduction step was obtained. Percentage uncertainties in these mean rate constants were calculated using

Equation 4 below, where $k_{n(\mathrm{min})}$ and $k_{n(\mathrm{max})}$ are the minimum and maximum rate constants across all replicates for reduction step n and $k_{M(n)}$ is the mean rate constant for reduction step n.

$$\% \text{ Uncertainty} = \frac{\left(\frac{(k_{n(\max)} - k_{n(min)})}{2}\right)}{k_{M(n)}} \times 100 \qquad \text{(Equation 4)}$$

While all calculated uncertainties were found to be 5% or less, we conservatively associate an error of ± 10% on all reaction rate constants presented herein involving reactions of precursor ions of charge states 13^+ to 2^+. The electrochemical reaction rate increases as the charge state of the precursor ion increases, and as a result, cages with higher charge states (14^+ to 16^+) react with fluoranthene radical anions at rates approaching the temporal resolution of the instrumentation available. Accordingly, we estimate the error in rate constants with these precursor ions to be as high as 20%.

Table S2: Rate constants (k_n, as defined above), mean rate constants in s^{-1} ($\times 10^{-3}$) and their uncertainties from repeated experiments involving the sequential reductions of Ni-**1**$(OTf)_5^{11+}$ (Table S7, Figure S19) and $C_{60}\subset$Ni-**1**$(OTf)_5^{11+}$ (Table S8, Figure S20). Experiments carried out over the period of 10 days. Absolute uncertainty in mean given within parentheses.

**Precursor ion**	k_1	k_2	k_3	k_4	k_5	k_6	k_7	k_8	k_9	k_{10}
Ni-**1**$(OTf)_5^{11+}$	0.3038	0.2724	0.2188	0.1854	0.1468	0.1149	0.0916	0.0635	0.0423	0.0249
	0.3140	0.2719	0.2339	0.1864	0.1510	0.1152	0.0905	0.0653	0.0443	0.0261
	0.3021	0.2831	0.2331	0.1890	0.1508	0.1183	0.0857	0.0646	0.0455	0.0255
	0.3094	0.2724	0.2309	0.1891	0.1536	0.1231	0.0869	0.0649	0.0445	0.0261
	0.3024	0.2773	0.2346	0.1943	0.1534	0.1167	0.0872	0.0669	0.0445	0.0269
	0.3090	0.2726	0.2258	0.1935	0.1534	0.1190	0.0881	0.0658	0.0445	0.0252
	0.3126	0.2814	0.2201	0.1935	0.1579	0.1254	0.0904	0.0672	0.0450	-
Mean	0.308(6)	0.276(6)	0.228(8)	0.190(4)	0.152(6)	0.119(5)	0.089(3)	0.066(2)	0.044(2)	0.026(1)
% Uncertainty	1.9	2.0	3.5	2.3	3.6	4.4	3.3	2.8	3.6	3.9

**Precursor ion**	k_1	k_2	k_3	k_4	k_5	k_6	k_7	k_8	k_9
$C_{60}\subset$Ni-**1**$(OTf)_5^{11+}$	0.3088	0.2704	0.2266	0.1859	0.1489	0.1202	0.0894	0.0626	0.0428
	0.3024	0.2899	0.2364	0.1898	0.1485	0.1161	0.0889	0.0654	0.0450
	0.3040	0.2892	0.2306	0.1894	0.1510	0.1152	0.0880	0.0676	0.0440
	0.3068	0.2777	0.2339	0.1910	0.1532	0.1202	0.0917	0.0645	0.0442
	0.3169	0.2788	0.2280	0.1852	0.1546	0.1238	0.0920	0.0652	0.0429
	0.3029	0.2906	0.2261	0.1866	0.1530	0.1244	0.0894	0.0663	0.0436
	0.3080	0.2977	0.2280	0.1905	0.1637	0.1246	0.0893	0.0668	0.0463
Mean	0.307(7)	0.28(1)	0.230(5)	0.188(3)	0.153(8)	0.120(5)	0.090(2)	0.066(3)	0.044(2)
% Uncertainty	2.4	4.8	2.2	1.5	5.0	3.9	2.2	3.8	4.0

To validate this method, further experimentation was carried out wherein each successive reduced species was re-isolated and reacted with $FL^{-•}$. In this way, the reaction rate at each reduction step could be modelled using a single exponential equation. Rate constants obtained in this way (Table S3) were found to fall within experimental error of those calculated using Equation 3.

Table S3: Comparison of rate constants (k_n, as defined above, in s^{-1} ($\times 10^{-3}$)) for the 7-step sequential reduction of Ni-**1**$(OTf)_7^{9+}$ (Table S9, Figure S21) obtained using Equation 3 *vs* re-isolating each successive species for reaction with $FL^{-•}$ (i.e. k_1 is the rate constant for the reaction Ni-**1**$(OTf)_7^{9+}$ + $FL^{-•}$ → Ni-**1**$(OTf)_7^{8+•}$ + FL). Absolute uncertainties provided in parentheses.

**Calculation Method**	k_1	k_2	k_3	k_4	k_5	k_6	k_7
Equation 3	0.22(2)	0.19(2)	0.16(2)	0.11(1)	0.10(1)	0.064(6)	0.045(5)
Re-isolation (Eq. 2)	0.22(2)	0.19(2)	0.15(2)	0.11(1)	0.087(9)	0.066(7)	0.047(5)

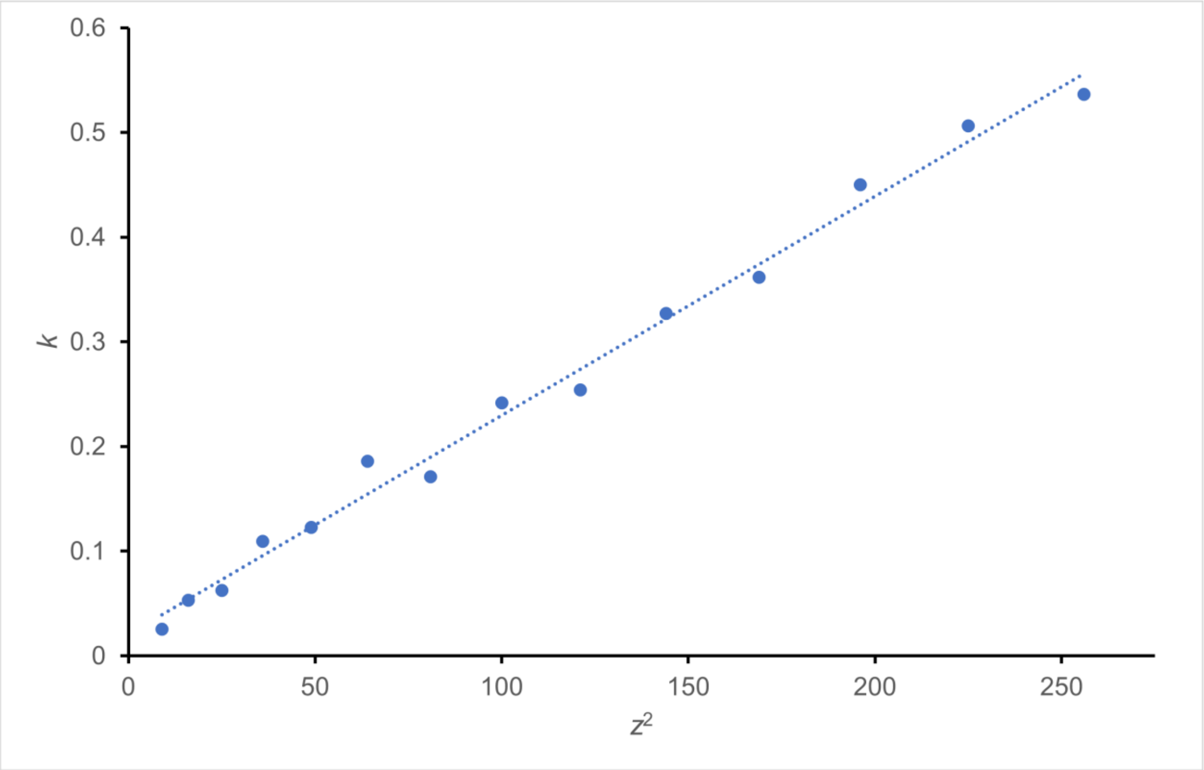

Figure S11: Comparison of calculated rate constants for the sequential reduction of Ni-**1**$^{16+}$ with z^2 (R^2 = 0.9937)

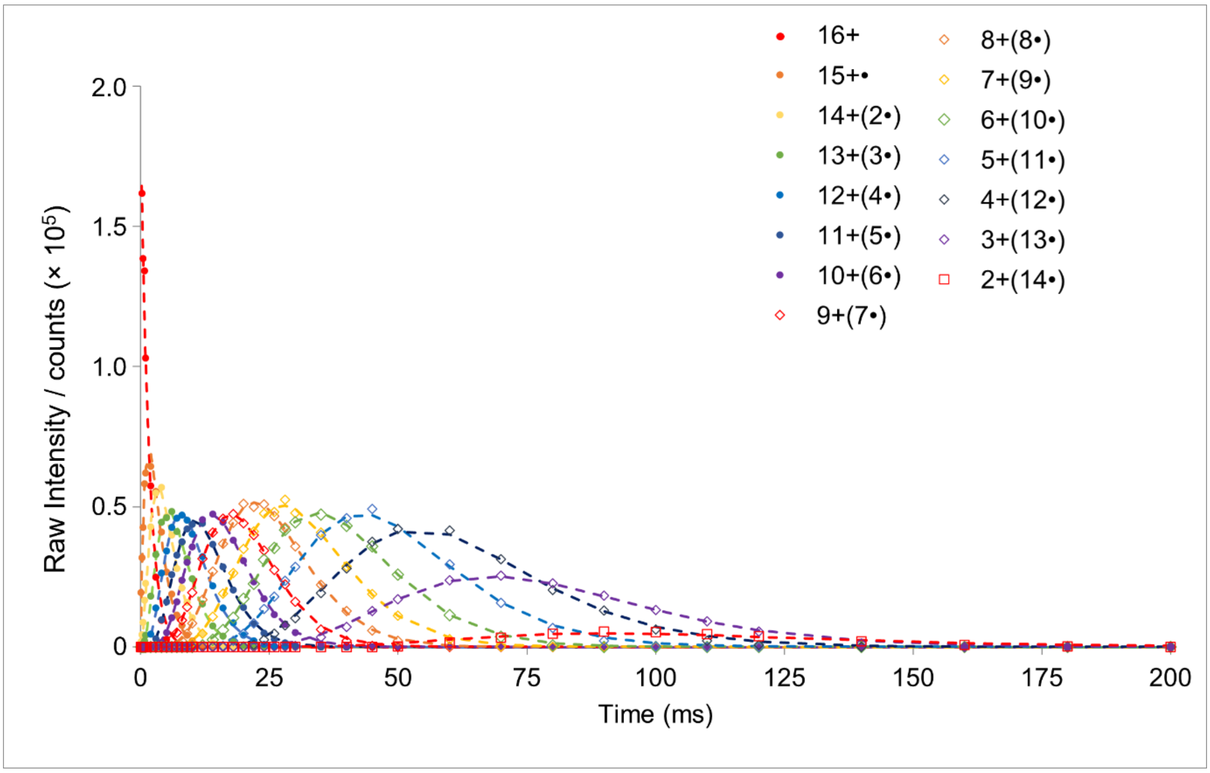

Figure S12: Modelling of the growth and decay kinetics of the products of reaction of C_{60}⊂Ni-**1**$^{16+}$ with $FL^{-\bullet}$ over 200 ms using Equation 3.

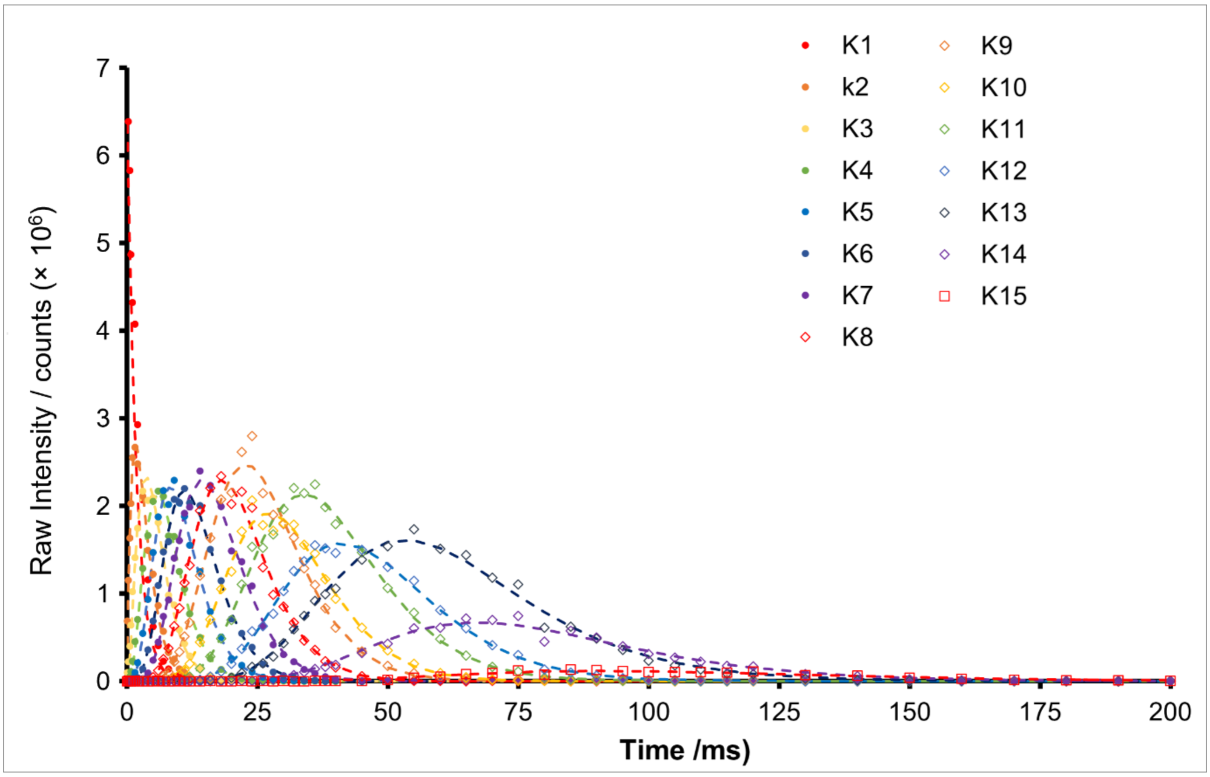

Figure S13: Modelling of the growth and decay kinetics of the products of reaction of H_2-**1**$^{16+}$ with $FL^{-\bullet}$ over 200 ms using Equation 3.

Cyclic Voltammetry

Cyclic voltammetry was performed on a 0.1 mM solution of cage Ni-**1** with a Biologic SP-150 potentiostat under an argon atmosphere in anhydrous 1:4 acetonitrile:dimethyl formamide and 0.1M concentration of $TBAPF_6$ salt. All measurements were carried out in *ca.* 1 mL sample aliquots using a standard 3-electrode electrochemical cell. A glassy carbon electrode with a surface area of 0.07069 cm^2 was used as the main working electrode with rigorous cleaning in between each experiment. An aluminium fibre pad with a slurry of aluminium oxide polishing powder was used for gentle surface abrasion for surface particulates removal and subsequently washed with Milli-Q water and finally with acetone. A solid platinum wire counter electrode and an eDAQ leakless Ag/AgCl reference was used, rinsed with acetonitrile before use. Ferrocene was used as an external reference standard.

The cathodic sweep revealed a series of irreversible reduction processes indicating some degree of communication between the redox centres. Interestingly one quasi-reversible redox couple was observed in this range (−1.76 V *vs.* Fc/Fc^+) which we tentatively attribute to the single electron reduction/oxidation of the Ni porphyrin moiety based on related tetra-substituted nickel(II) porphyrins reduction potentials.[11] These results indicate that while a series of multiply reduced species akin to those observed in the gas phase can be formed in solution, investigations of the properties of the cage at each individual redox state are more accessible in the latter where purification through mass-selection is possible.

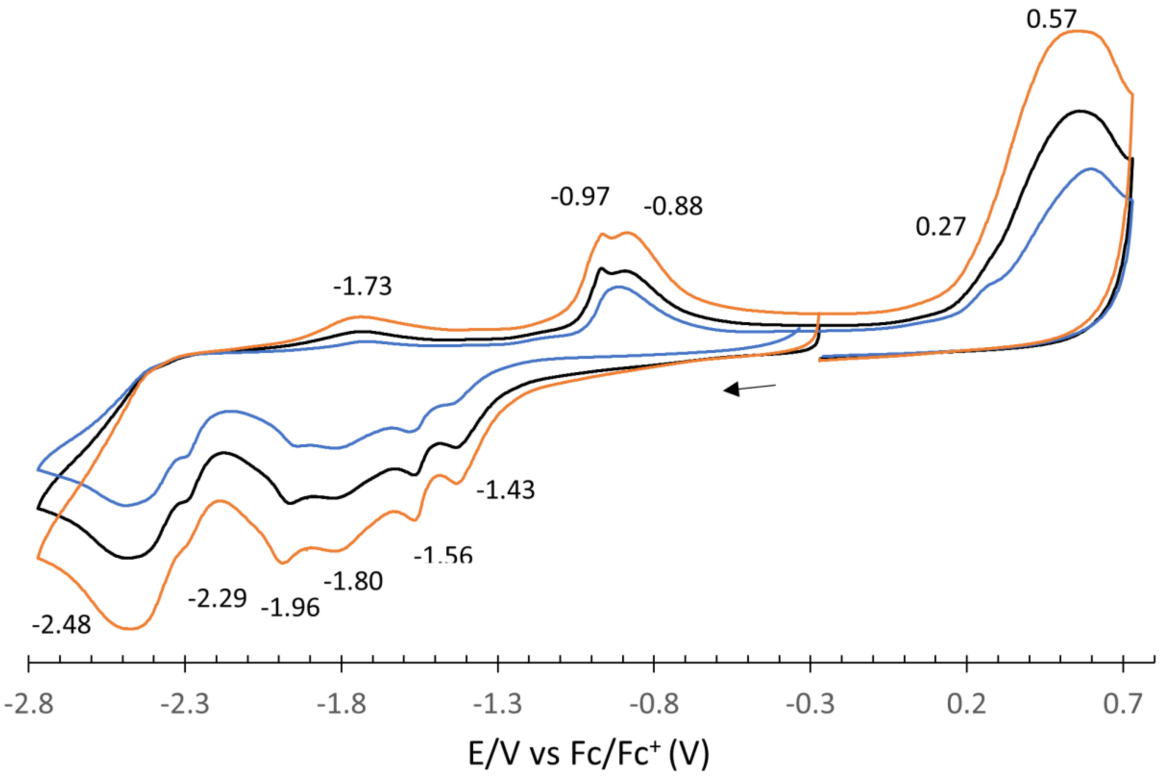

Figure S14: Cyclic voltammogram of Ni-**1** in 0.1 mM $n Bu_4NPF_6$ / 1:4 dimethylformamide:acetonitrile scanned at 25 (blue), 50 (black) and 100 (orange) mV s^{-1}. The arrow indicates the direction of the scan. Superscript numbers indicate the potential of observed redox peak. Oxidation peak at 0.57 V is believed

to be oxidation of Fe(II) →Fe(III) leading to irreversible collapse of the complex and passivation of the electrode surface.

Dioxygen Reactivity Investigations

Following reduction of an ion to the desired charge state with $FL^{-\bullet}$, the reduced ion was re-isolated using an isolation window of 15 Da and held in the ion trap with no supplemental collision energy for 200 to 2000 ms. After this time, the reaction products were scanned out and analysed in the Orbitrap.

Differences between observed isotopic distributions for O_2 reaction products (Figure S15, S22) and the expected naturally occurring distributions are attributed to lower signal intensity, as well as the use of two subsequent mass selection processes (*i.e.*, an MS^3 experiment), which may skew the observed isotopic pattern away from the naturally occurring distribution.

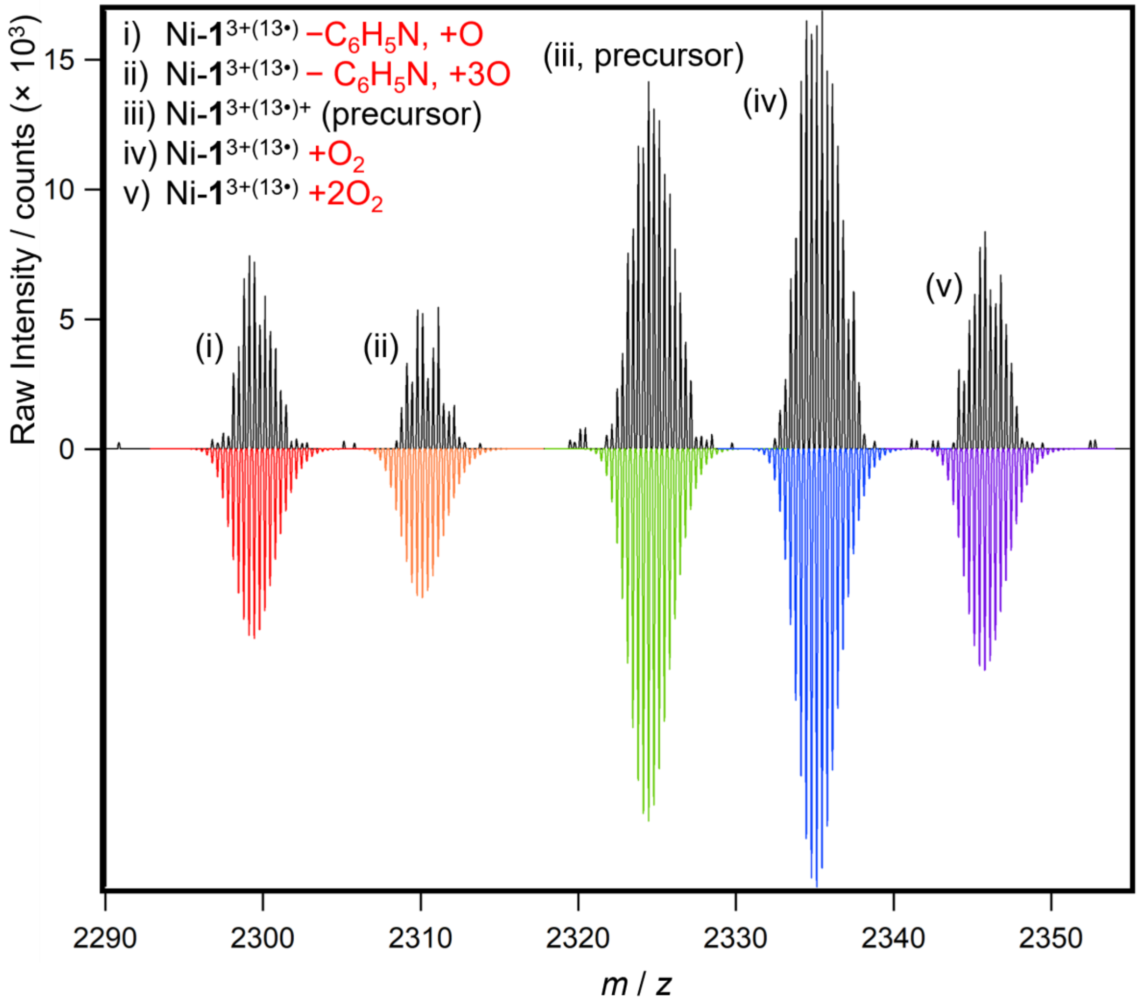

Figure S15: Comparison of experimental (top) and simulated isotopic distributions (bottom) for reaction products present after holding reduced ions Ni-**1**$^{3+(13\bullet)}$ in the ion trap with O_2 for 200 ms (see also in Figure 4d).

Comparisons of Experimental Mass Spectrometry Data to Theoretical Simulations

Table S4: Experimentally measured mass-to-charge ratios (*m*/*z*) of product ions (base peak of isotopic distribution) resulting from electrochemical reduction of cage Ni-**1** with $FL^{-\bullet}$ shown in Figure 3a.

Assignment	Reaction Time / ms	Experimental *m/z*	Chemical Formula	Theoretical *m/z*	Δ / ppm
Ni-$\mathbf{1}^{16+}$	1	435.8363	$C_{408}H_{264}N_{72}Fe_8Ni_6^{16+}$	435.8361	0.5
Ni-$\mathbf{1}^{15+\bullet}$	1	464.8921	$C_{408}H_{264}N_{72}Fe_8Ni_6^{15+\bullet}$	464.8918	0.6
Ni-$\mathbf{1}^{14+(2\bullet)}$	1	498.0985	$C_{408}H_{264}N_{72}Fe_8Ni_6^{14+(2\bullet)}$	498.0984	0.2
Ni-$\mathbf{1}^{13+(3\bullet)}$	1	563.4136	$C_{408}H_{264}N_{72}Fe_8Ni_6^{13+(3\bullet)}$	563.4137	0.2
Ni-$\mathbf{1}^{12+(4\bullet)}$	12	581.1150	$C_{408}H_{264}N_{72}Fe_8Ni_6^{12+(4\bullet)}$	581.1149	0.2
Ni-$\mathbf{1}^{11+(5\bullet)}$	12	633.9434	$C_{408}H_{264}N_{72}Fe_8Ni_6^{11+(5\bullet)}$	633.9436	0.3
Ni-$\mathbf{1}^{10+(6\bullet)}$	12	697.3379	$C_{408}H_{264}N_{72}Fe_8Ni_6^{10+(6\bullet)}$	697.3380	0.1
Ni-$\mathbf{1}^{9+(7\bullet)}$	12	774.8198	$C_{408}H_{264}N_{72}Fe_8Ni_6^{9+(7\bullet)}$	774.8201	0.4
Ni-$\mathbf{1}^{8+(8\bullet)}$	30	871.6722	$C_{408}H_{264}N_{72}Fe_8Ni_6^{8+(8\bullet)}$	871.6721	0.1
Ni-$\mathbf{1}^{7+(9\bullet)}$	50	996.1967	$C_{408}H_{264}N_{72}Fe_8Ni_6^{7+(9\bullet)}$	996.1974	0.7
Ni-$\mathbf{1}^{6+(10\bullet)}$	50	1162.2294	$C_{408}H_{264}N_{72}Fe_8Ni_6^{6+(10\bullet)}$	1162.2304	0.9
Ni-$\mathbf{1}^{5+(11\bullet)}$	50	1394.6760	$C_{408}H_{264}N_{72}Fe_8Ni_6^{5+(11\bullet)}$	1394.6766	0.4
Ni-$\mathbf{1}^{4+(12\bullet)}$	50	1743.3448	$C_{408}H_{264}N_{72}Fe_8Ni_6^{4+(12\bullet)}$	1743.3459	0.6
Ni-$\mathbf{1}^{3+(13\bullet)}$	90	2324.4586	$C_{408}H_{264}N_{72}Fe_8Ni_6^{3+(13\bullet)}$	2324.4614	1.2
Ni-$\mathbf{1}^{2+(14\bullet)}$	110	3486.6858	$C_{408}H_{264}N_{72}Fe_8Ni_6^{2+(14\bullet)}$	3486.6924	1.9

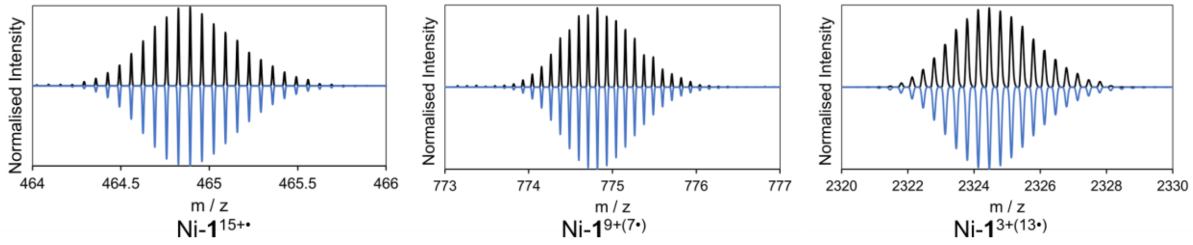

Figure S16: Comparisons of experimental isotopic distributions for selected electrochemically reduced ions of Ni-$\mathbf{1}^{16+}$ (experimental top, theoretical bottom).

Table S5: Experimentally measured mass-to-charge ratios (*m*/*z*) of product ions (base peak of isotopic distribution) resulting from electrochemical reduction of cage C_{60}⊂Ni-**1** with $FL^{-•}$ shown in Figure S11.

Assignment	Reaction Time / ms	Experimental *m/z*	Chemical Formula	Theoretical *m/z*	Δ / ppm
C_{60}⊂Ni-**1**$^{16+}$	1	480.8364	$C_{468}H_{264}N_{72}Fe_8Ni_6^{16+}$	480.8362	0.4
C_{60}⊂Ni-**1**$^{15+•}$	1	512.8920	$C_{468}H_{264}N_{72}Fe_8Ni_6^{15+•}$	512.8920	0.0
C_{60}⊂Ni-**1**$^{14+(2•)}$	1	549.5271	$C_{468}H_{264}N_{72}Fe_8Ni_6^{14+(2•)}$	549.5272	0.2
C_{60}⊂Ni-**1**$^{13+(3•)}$	1	591.7982	$C_{468}H_{264}N_{72}Fe_8Ni_6^{13+(3•)}$	591.7985	0.5
C_{60}⊂Ni-**1**$^{12+(4•)}$	12	641.1154	$C_{468}H_{264}N_{72}Fe_8Ni_6^{12+(4•)}$	641.1151	0.5
C_{60}⊂Ni-**1**$^{11+(5•)}$	12	699.3984	$C_{468}H_{264}N_{72}Fe_8Ni_6^{11+(5•)}$	699.3984	0.0
C_{60}⊂Ni-**1**$^{10+(6•)}$	12	769.3383	$C_{468}H_{264}N_{72}Fe_8Ni_6^{10+(6•)}$	769.3383	0.0
C_{60}⊂Ni-**1**$^{9+(7•)}$	12	854.8205	$C_{468}H_{264}N_{72}Fe_8Ni_6^{9+(7•)}$	854.8204	0.1
C_{60}⊂Ni-**1**$^{8+(8•)}$	30	961.6728	$C_{468}H_{264}N_{72}Fe_8Ni_6^{8+(8•)}$	961.6730	0.2
C_{60}⊂Ni-**1**$^{7+(9•)}$	50	1099.0542	$C_{468}H_{264}N_{72}Fe_8Ni_6^{7+(9•)}$	1099.0549	0.6
C_{60}⊂Ni-**1**$^{6+(10•)}$	50	1282.2308	$C_{468}H_{264}N_{72}Fe_8Ni_6^{6+(10•)}$	1282.2303	0.4
C_{60}⊂Ni-**1**$^{5+(11•)}$	50	1538.6771	$C_{468}H_{264}N_{72}Fe_8Ni_6^{5+(11•)}$	1538.6773	0.1
C_{60}⊂Ni-**1**$^{4+(12•)}$	50	1923.3468	$C_{468}H_{264}N_{72}Fe_8Ni_6^{4+(12•)}$	1923.3465	0.2
C_{60}⊂Ni-**1**$^{3+(13•)}$	90	2564.4626	$C_{468}H_{264}N_{72}Fe_8Ni_6^{3+(13•)}$	2564.4621	0.2
C_{60}⊂Ni-**1**$^{2+(14•)}$	110	3846.7208	$C_{468}H_{264}N_{72}Fe_8Ni_6^{2+(14•)}$	3846.7080	3.3

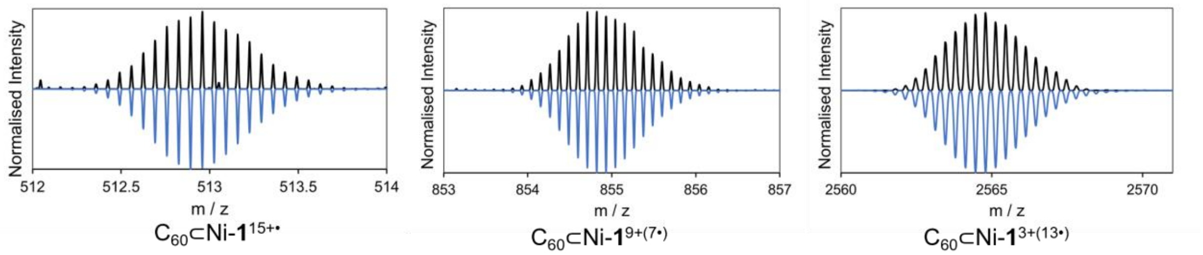

Figure S17: Comparisons of experimental isotopic distributions for selected electrochemically reduced ions of C_{60}⊂Ni-**1**$^{16+}$ (experimental top, theoretical bottom).

Table S6: Experimentally measured mass-to-charge ratios (*m*/*z*) of product ions (base peak of isotopic distribution) resulting from electrochemical reduction of cage H_2-**1** with $FL^{-\bullet}$ shown in Figure S13.

Assignment	Reaction Time / ms	Experimental *m/z*	Chemical Formula	Theoretical *m/z*	Δ / ppm
H_2-$\mathbf{1}^{16+}$	1	414.6159	$C_{408}H_{276}N_{72}Fe_8^{16+}$	414.6167	1.9
H_2-$\mathbf{1}^{15+\bullet}$	1	442.2567	$C_{408}H_{276}N_{72}Fe_8^{15+\bullet}$	442.2578	2.5
H_2-$\mathbf{1}^{14+(2\bullet)}$	1	473.8466	$C_{408}H_{276}N_{72}Fe_8^{14+(2\bullet)}$	473.8477	2.3
H_2-$\mathbf{1}^{13+(3\bullet)}$	1	510.2965	$C_{408}H_{276}N_{72}Fe_8^{13+(3\bullet)}$	510.2976	2.2
H_2-$\mathbf{1}^{12+(4\bullet)}$	12	552.8211	$C_{408}H_{276}N_{72}Fe_8^{12+(4\bullet)}$	552.8224	2.4
H_2-$\mathbf{1}^{11+(5\bullet)}$	12	603.0776	$C_{408}H_{276}N_{72}Fe_8^{11+(5\bullet)}$	603.0791	2.5
H_2-$\mathbf{1}^{10+(6\bullet)}$	12	663.3853	$C_{408}H_{276}N_{72}Fe_8^{10+(6\bullet)}$	663.3870	2.6
H_2-$\mathbf{1}^{9+(7\bullet)}$	12	737.0946	$C_{408}H_{276}N_{72}Fe_8^{9+(7\bullet)}$	737.0967	2.8
H_2-$\mathbf{1}^{8+(8\bullet)}$	30	829.2314	$C_{408}H_{276}N_{72}Fe_8^{8+(8\bullet)}$	829.2339	3.0
H_2-$\mathbf{1}^{7+(9\bullet)}$	50	947.6926	$C_{408}H_{276}N_{72}Fe_8^{7+(9\bullet)}$	947.6960	3.6
H_2-$\mathbf{1}^{6+(10\bullet)}$	50	1105.6415	$C_{408}H_{276}N_{72}Fe_8^{6+(10\bullet)}$	1105.6454	3.5
H_2-$\mathbf{1}^{5+(11\bullet)}$	50	1326.7701	$C_{408}H_{276}N_{72}Fe_8^{5+(11\bullet)}$	1326.7746	3.4
H_2-$\mathbf{1}^{4+(12\bullet)}$	50	1658.4645	$C_{408}H_{276}N_{72}Fe_8^{4+(12\bullet)}$	1658.4683	2.3
H_2-$\mathbf{1}^{3+(13\bullet)}$	90	2211.2865	$C_{408}H_{276}N_{72}Fe_8^{3+(13\bullet)}$	2211.2913	2.2
H_2-$\mathbf{1}^{2+(14\bullet)}$	110	3316.9352	$C_{408}H_{276}N_{72}Fe_8^{2+(14\bullet)}$	3316.9372	0.6

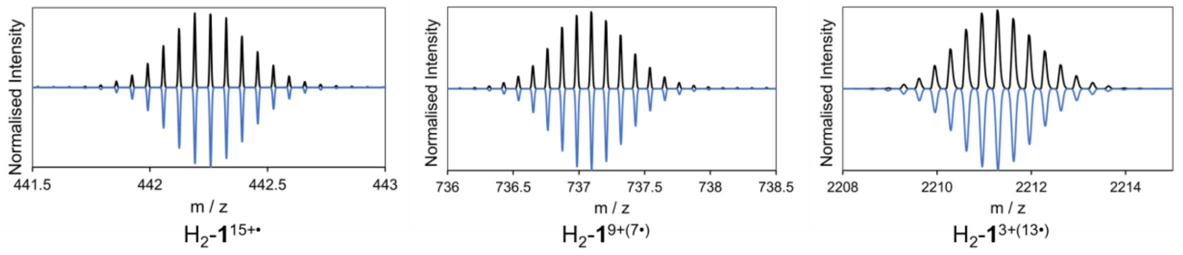

Figure S18: Comparisons of experimental isotopic distributions for selected electrochemically reduced ions of H_2-$\mathbf{1}^{16+}$ (experimental top, theoretical bottom).

Table S7: Experimentally measured mass-to-charge ratios (*m*/*z*) of product ions (base peak of isotopic distribution) resulting from electrochemical reduction of cage Ni-**1**$(OTf)_5^{11+}$ with $FL^{-\bullet}$.

**Assignment**	**Reaction Time / ms**	**Experimental *m*/*z***	**Chemical Formula**	**Theoretical *m*/*z***	**Δ / ppm**
Ni-**1**$(OTf)_5^{11+}$	1	701.6493	$C_{413}H_{264}N_{72}O_{15}F_{15}S_5Fe_8Ni_6^{11+}$	701.6491	0.3
Ni-**1**$(OTf)_5^{10+\bullet}$	1	771.8139	$C_{413}H_{264}N_{72}O_{15}F_{15}S_5Fe_8Ni_6^{10+\bullet}$	771.8140	0.1
Ni-**1**$(OTf)_5^{9+(2\bullet)}$	12	857.5713	$C_{413}H_{264}N_{72}O_{15}F_{15}S_5Fe_8Ni_6^{9+(2\bullet)}$	857.5712	0.1
Ni-**1**$(OTf)_5^{8+(3\bullet)}$	12	964.7675	$C_{413}H_{264}N_{72}O_{15}F_{15}S_5Fe_8Ni_6^{8+(3\bullet)}$	964.7677	0.2
Ni-**1**$(OTf)_5^{7+(4\bullet)}$	12	1102.5915	$C_{413}H_{264}N_{72}O_{15}F_{15}S_5Fe_8Ni_6^{7+(4\bullet)}$	1102.5917	0.2
Ni-**1**$(OTf)_5^{6+(5\bullet)}$	12	1286.3568	$C_{413}H_{264}N_{72}O_{15}F_{15}S_5Fe_8Ni_6^{6+(5\bullet)}$	1286.3571	0.2
Ni-**1**$(OTf)_5^{5+(6\bullet)}$	30	1543.6278	$C_{413}H_{264}N_{72}O_{15}F_{15}S_5Fe_8Ni_6^{5+(6\bullet)}$	1543.6286	0.5
Ni-**1**$(OTf)_5^{4+(7\bullet)}$	30	1929.5344	$C_{413}H_{264}N_{72}O_{15}F_{15}S_5Fe_8Ni_6^{4+(7\bullet)}$	1929.5359	0.8
Ni-**1**$(OTf)_5^{3+(8\bullet)}$	50	2572.7101	$C_{413}H_{264}N_{72}O_{15}F_{15}S_5Fe_8Ni_6^{3+(8\bullet)}$	2572.7147	1.8
Ni-**1**$(OTf)_5^{2+(9\bullet)}$	90	3859.0652	$C_{413}H_{264}N_{72}O_{15}F_{15}S_5Fe_8Ni_6^{2+(9\bullet)}$	3859.0723	1.8

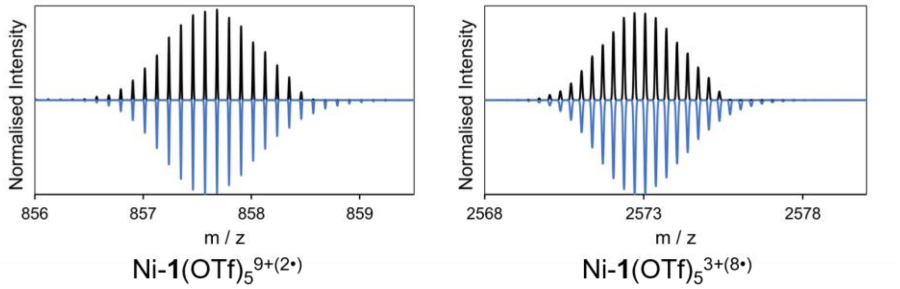

Figure S19: Comparisons of experimental isotopic distributions for selected electrochemically reduced ions of Ni-**1**$(OTf)_5^{11+}$ (experimental top, theoretical bottom).

Table S8: Experimentally measured mass-to-charge ratios (*m*/*z*) of product ions (base peak of isotopic distribution) resulting from electrochemical reduction of cage C_{60}⊂Ni-**1**$(OTf)_5^{11+}$ with FL$^{-\bullet}$.

Assignment	Reaction Time / ms	Experimental *m*/*z*	Chemical Formula	Theoretical *m*/*z*	Δ / ppm
C_{60}⊂Ni-**1**$(OTf)_5^{11+}$	1	767.1949	$C_{473}H_{264}N_{72}O_{15}F_{15}S_5Fe_8Ni_6^{11+}$	767.1947	0.3
C_{60}⊂Ni-**1**$(OTf)_5^{10+\bullet}$	1	843.9142	$C_{473}H_{264}N_{72}O_{15}F_{15}S_5Fe_8Ni_6^{10+\bullet}$	843.9142	0.0
C_{60}⊂Ni-**1**$(OTf)_5^{9+(2\bullet)}$	12	937.6822	$C_{473}H_{264}N_{72}O_{15}F_{15}S_5Fe_8Ni_6^{9+(2\bullet)}$	937.6825	0.3
C_{60}⊂Ni-**1**$(OTf)_5^{8+(3\bullet)}$	12	1054.8924	$C_{473}H_{264}N_{72}O_{15}F_{15}S_5Fe_8Ni_6^{8+(3\bullet)}$	1054.8929	0.5
C_{60}⊂Ni-**1**$(OTf)_5^{7+(4\bullet)}$	12	1205.5912	$C_{473}H_{264}N_{72}O_{15}F_{15}S_5Fe_8Ni_6^{7+(4\bullet)}$	1205.5920	0.7
C_{60}⊂Ni-**1**$(OTf)_5^{6+(5\bullet)}$	12	1406.5238	$C_{473}H_{264}N_{72}O_{15}F_{15}S_5Fe_8Ni_6^{6+(5\bullet)}$	1406.5240	0.1
C_{60}⊂Ni-**1**$(OTf)_5^{5+(6\bullet)}$	30	1687.8277	$C_{473}H_{264}N_{72}O_{15}F_{15}S_5Fe_8Ni_6^{5+(6\bullet)}$	1687.8290	0.8
C_{60}⊂Ni-**1**$(OTf)_5^{4+(7\bullet)}$	30	2109.7848	$C_{473}H_{264}N_{72}O_{15}F_{15}S_5Fe_8Ni_6^{4+(7\bullet)}$	2109.7863	0.7
C_{60}⊂Ni-**1**$(OTf)_5^{3+(8\bullet)}$	50	2813.0463	$C_{473}H_{264}N_{72}O_{15}F_{15}S_5Fe_8Ni_6^{3+(8\bullet)}$	2813.0486	0.8

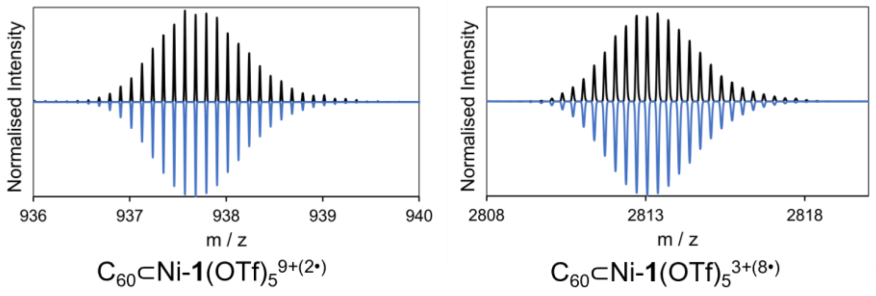

Figure S20: Comparisons of experimental isotopic distributions for selected electrochemically reduced ions of C_{60}⊂Ni-**1**$(OTf)_5^{11+}$ (experimental top, theoretical bottom).

Table S9 Experimentally measured mass-to-charge ratios (*m*/*z*) of product ions (base peak of isotopic distribution) resulting from electrochemical reduction of cage Ni-**1**$(OTf)_7^{9+}$ with $FL^{-•}$.

**Assignment**	**Reaction Time / ms**	**Experimental *m*/*z***	**Chemical Formula**	**Theoretical *m*/*z***	**Δ / ppm**
Ni-**1**$(OTf)_7^{9+}$	1	890.6735	$C_{415}H_{264}N_{72}O_{21}F_{21}S_7Fe_8Ni_6^{9+}$	890.6717	2.0
Ni-**1**$(OTf)_7^{8+•}$	1	1002.1323	$C_{415}H_{264}N_{72}O_{21}F_{21}S_7Fe_8Ni_6^{8+•}$	1002.1306	1.7
Ni-**1**$(OTf)_7^{7+(2•)}$	12	1145.2937	$C_{415}H_{264}N_{72}O_{21}F_{21}S_7Fe_8Ni_6^{7+(2•)}$	1145.2922	1.3
Ni-**1**$(OTf)_7^{6+(3•)}$	12	1336.1764	$C_{415}H_{264}N_{72}O_{21}F_{21}S_7Fe_8Ni_6^{6+(3•)}$	1336.1743	1.6
Ni-**1**$(OTf)_7^{5+(4•)}$	12	1603.4123	$C_{415}H_{264}N_{72}O_{21}F_{21}S_7Fe_8Ni_6^{5+(4•)}$	1603.4093	1.9
Ni-**1**$(OTf)_7^{4+(5•)}$	30	2004.2625	$C_{415}H_{264}N_{72}O_{21}F_{21}S_7Fe_8Ni_6^{4+(5•)}$	2004.2617	0.4
Ni-**1**$(OTf)_7^{3+(6•)}$	50	2672.3509	$C_{415}H_{264}N_{72}O_{21}F_{21}S_7Fe_8Ni_6^{3+(6•)}$	2672.3492	0.6

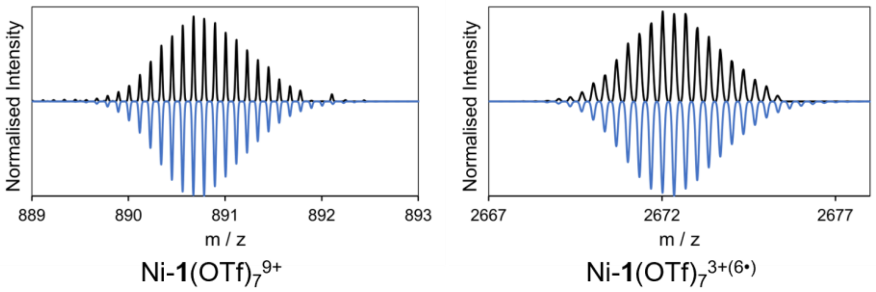

Figure S21: Comparisons of experimental isotopic distributions for selected electrochemically reduced ions of Ni-**1**$(OTf)_7^{9+}$ (experimental top, theoretical bottom).

Table S10: Experimentally measured mass-to-charge ratios (*m*/*z*) of product ions (base peak of isotopic distribution) resulting from reaction of reduced cages with O_2 as shown in Figure 4.

**Assignment**	**Experimental *m/z***	**Chemical Formula**	**Theoretical *m/z***	**Δ / ppm**
Ni-**1**$^{3+(13\bullet)}\cdot O_2$	2335.1270	$C_{408}H_{264}N_{72}O_2Fe_8Ni_6{}^{3+(13\bullet)}$	2335.1247	1.0
Ni-**1**$^{3+(13\bullet)}\cdot 2O_2$	2345.8018	$C_{408}H_{264}N_{72}O_4Fe_8Ni_6{}^{3+(13\bullet)}$	2345.7986	1.4
Ni-**1**$^{3+(13\bullet)}$ – 6C5HN + O	2299.4456	$C_{402}H_{259}N_{72}O_1Fe_8Ni_6{}^{3+(13\bullet)}$	2299.4562	4.6
Ni-**1**$^{3+(13\bullet)}$ – 6C5HN + 3O	2310.1088	$C_{402}H_{259}N_{72}O_3Fe_8Ni_6{}^{3+(13\bullet)}$	2310.1195	4.6
$C_{60}\subset$Ni-**1**$^{3+(13\bullet)}\cdot O_2$	2575.1442	$C_{468}H_{264}N_{72}O_2Fe_8Ni_6{}^{3+(13\bullet)}$	2575.1353	3.5
$C_{60}\subset$Ni-**1**$^{3+(13\bullet)}\cdot 2O_2$	2586.1410	$C_{468}H_{264}N_{72}O_2Fe_8Ni_6{}^{3+(13\bullet)}$	2586.1319	3.5

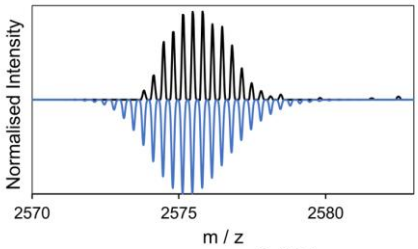

$C_{60}\subset$Ni-**1**+$O_2{}^{3+(13\bullet)}$

Figure S22: Comparison of experimental isotopic distribution for ions of $C_{60}\subset$Ni-**1**$^{3+(13\bullet)}\cdot 2O_2$ (experimental top, theoretical bottom).

References

[1] W. Meng, B. Breiner, K. Rissanen, J. D. Thoburn, J. K. Clegg, J. R. Nitschke, *Angew. Chem., Int. Ed.* **2011**, *50*, 3479-3483.

[2] a) J. E. P. Syka, J. J. Coon, M. J. Schroeder, J. Shabanowitz, D. F. Hunt, *Proceedings of the National Academy of Sciences of the United States of America* **2004**, *101*, 9528-9533; b) E. D. A. Michalski, O. Lange, E. Denisov, D. Nolting, M. Müller, R. Viner, J. Schwartz, P. Remes, M. Belford, J.-J. Dunyach, J. Cox, S. Horning, M. Mann, A. Makarov. , *Mol. Cell. Proteomics* **2012**, *11*, O111.013698.

[3] A. A. Shvartsburg, R. D. Smith, *Anal. Chem.* **2008**, *80*, 9689-9699.

[4] E. Hanozin, B. Mignolet, J. Martens, G. Berden, D. Sluysmans, A-S. Duwez, J. F. Stoddart, G. Eppe, J. Oomens, E. D. Pauw, D. Morsa, *Angew. Chem., Int. Ed.* **2021**, *60*, 10049-10055.

[5] B. T. Ruotolo, J. L. P. Benesch, A. M. Sandercock, S.-J. Hyung, C. V. Robinson, *Nat. Protoc.* **2008**, *3*, 1139–1152.

[6] M. F. Bush, Z. Hall, K. Giles, J. Hoyes, C. V. Robinson, B. T. Ruotolo, *Anal. Chem.* **2010**, *82*, 9557–9565.

[7] a) S. A. McLuckey, J. L. Stephenson, *Mass. Spec. Rev.* **1998**, *17*, 369-407; b) J. L. Stephenson, S. A. McLuckey, *J. Am. Chem. Soc.* **1996**, *118*, 7390-7397.

[8] a) L. M. Raff, *Principles of Physical Chemistry, Vol. 1st ed.*, Prentice Hall, New Jersey, **2001**; b) P. D. Compton, J. V. Strukl, D. L. Bai, J. Shabanowitz, D. F. Hunt, *Anal. Chem.* **2012**, *84*, 1781-1785; c) A. W. D. Nolting, in *60th ASMS Conference on Mass Spectrometry and Allied Topics*, Vancouver BC, **2012**; d) C. M. Rose, M. J. P. Rush, N. M. Riley, A. E. Merrill, N. W. Kwiecien, D. D. Holden, C. Mullen, M. S. Westphall, J. J. Coon, *J. Am. Soc. Mass Spectrom.* **2015**, *26*, 1848-1857.

[9] D. L. Marshall, B. L. J. Poad, E. T. Luis, R. A. Da Silva Rodrigues, S. J. Blanksby, K. M. Mullen, *Chem. Commun.* **2020**, *56*, 13575-13578.

[10] R. C. Bailey, G. S. Eadie, F. H. Schmidt, *Biometrics* **1974**, *30*, 67-75.

[11] D. Chang, T. Malinski, A. Ulman, K. M. Kadish, *Inorg. Chem.* **1984**, *23*, 817-824.
